# Linking Physical Activity to Breast Cancer via Inflammation, Part 2: The Effect of Inflammation on Breast Cancer Risk

**DOI:** 10.1158/1055-9965.EPI-22-0929

**Published:** 2023-03-03

**Authors:** Makayla W.C. Lou, Ann E. Drummond, Christopher T.V. Swain, Roger L. Milne, Dallas R. English, Kristy A. Brown, Eline H. van Roekel, Tina L. Skinner, Melissa M. Moore, Tom R. Gaunt, Richard M. Martin, Sarah J. Lewis, Brigid M. Lynch

**Affiliations:** 1 Cancer Epidemiology Division, Cancer Council Victoria, Victoria, Australia.; 2 Centre for Epidemiology and Biostatistics, Melbourne School of Population and Global Health, The University of Melbourne, Melbourne, Australia.; 3 Precision Medicine, School of Clinical Sciences at Monash Health, Monash University, Melbourne, Australia.; 4 Department of Medicine, Weill Cornell Medicine, New York, New York.; 5 Department of Epidemiology, GROW School for Oncology and Developmental Biology, Maastricht University, Maastricht, the Netherlands.; 6 School of Human Movement and Nutrition Sciences, Faculty of Health and Behavioural Sciences, University of Queensland, Brisbane, Australia.; 7 Medical Oncology, St Vincent's Hospital, Melbourne, Australia.; 8 Department of Medicine, The University of Melbourne, Melbourne, Australia.; 9 Bristol Medical School, University of Bristol, Bristol, United Kingdom.; 10 NIHR Biomedical Research Centre at University Hospitals Bristol and Weston NHS Foundation Trust and the University of Bristol, Bristol, United Kingdom.; 11 Physical Activity Laboratory, Baker Heart and Diabetes Institute, Melbourne, Australia.

## Abstract

This review synthesized and appraised the evidence for an effect of inflammation on breast cancer risk. Systematic searches identified prospective cohort and Mendelian randomization studies relevant to this review. Meta-analysis of 13 biomarkers of inflammation were conducted to appraise the evidence for an effect breast cancer risk; we examined the dose–response of these associations. Risk of bias was evaluated using the ROBINS-E tool and the quality of evidence was appraised with Grading of Recommendations Assessment, Development, and Evaluation. Thirty-four observational studies and three Mendelian randomization studies were included. Meta-analysis suggested that women with the highest levels of C-reactive protein (CRP) had a higher risk of developing breast cancer [risk ratio (RR) = 1.13; 95% confidence interval (CI), 1.01–1.26] compared with women with the lowest levels. Women with highest levels of adipokines, particularly adiponectin (RR = 0.76; 95% CI, 0.61–0.91) had a reduced breast cancer risk, although this finding was not supported by Mendelian randomization analysis. There was little evidence of an effect of cytokines, including TNFα and IL6, on breast cancer risk. The quality of evidence for each biomarker ranged from very low to moderate. Beyond CRP, the published data do not clearly support the role of inflammation in the development of breast cancer.

## Introduction

Observational evidence suggests that physical activity has a protective effect on breast cancer risk (1, 2). The underlying mechanisms are yet to be clarified but may involve inflammation (1, 2). However, the certainty of a physical activity — inflammation — breast cancer pathway cannot be established without systematic synthesis and appraisal of the evidence for an effect of inflammation on breast cancer risk.

Our current understanding of the role of inflammation in carcinogenesis is underpinned by evidence from *in vivo* and *ex vivo* studies that implicate inflammatory biomarkers in all stages of tumor development (3). Inflammatory signals induce an elevation of mutagenic reactive oxygen species which cause cellular oxidative stress and DNA damage (4). Genomic instability allows budding tumor cells to acquire mutations, while a competent immune system places pressure on cells to keep mutations that favor survival (5). As cancer cells proliferate, the secretion of cytokines facilitates cancer cell growth and angiogenesis (6).

Some observational studies suggest that increased levels of circulating inflammatory biomarkers may increase breast cancer risk (7). C-reactive protein (CRP), a commonly measured, nonspecific biomarker of inflammation, is the biomarker that has been most frequently studied in the context of breast cancer development (8). Some observational studies have also reported that long-term users of NSAIDs have a reduced risk of breast cancer (9). Increased inflammatory changes in previously healthy breast tissue are also associated with breast cancer risk (10). While existing systematic reviews on CRP and breast cancer risk have generally found positive associations (8, 11), other inflammatory biomarkers and breast cancer development have not been carefully studied. Kehm and colleagues recently conducted a systematic review of inflammatory biomarkers and breast cancer risk but concluded that evidence from markers other than CRP is limited (7).

Despite a compelling biological hypothesis and evidence for immunomodulatory effects induced by physical activity (12), epidemiologic findings on the role of circulating inflammatory biomarkers on breast cancer risk have not been rigorously evaluated. This review uses the World Cancer Research Fund (WCRF) International and University of Bristol causal evidence synthesis framework for conducting systematic reviews of mechanistic pathways for exposure–cancer associations (1). Our previous systematic reviews addressed the impact of physical activity on the sex steroid hormone (13, 14) and insulin signaling pathways (15, 16) and how these effects may alter breast cancer risk. The first part of this two-part systematic review investigated the impact of physical activity on the production of inflammatory biomarkers (12). Here, we synthesize and appraise the evidence that circulating inflammatory biomarkers affect breast cancer risk.

## Materials and Methods

Details on the methods for this review have been published in a protocol paper (1) and registered on PROSPERO (CRD42020165689). Briefly, systematic searches for keywords and Medical Subject Headings were conducted in MEDLINE (Ovid) and EMBASE (Ovid) until 23 February 2021. The search strategy can be found in Supplementary Methods and Materials (Supplementary Table S1). Eligible studies had a prospective design and examined the association between at least one inflammatory biomarker and breast cancer incidence. Studies conducted in a cohort of patients with a diagnosed medical condition (such as allergy and autoimmune conditions) were excluded. Study participants were required to be post-menarche. The inflammatory biomarkers of interest were previously identified and reported in our protocol paper (1). These were CRP, TNFα, leptin, adiponectin, IL1, IL1β, IL6, IL8, IL10, IL13, IFNγ, chemokine ligand 2 (CCL2), and prostaglandins.

Two authors (M.W.C. Lou and A.E. Drummond) independently screened the titles and abstracts; where there was consensus that studies were not relevant, they were excluded. Two authors (M.W.C. Lou and A.E. Drummond) then reviewed the full text of remaining papers; studies were included on the basis of consensus. Data were extracted into pre-piloted tables and summarized descriptively. For biomarkers with results reported from at least three separate studies (excluding Mendelian randomization studies), random-effects meta-analysis was conducted to estimate the effect size (risk ratio) for the highest category of biomarker compared with the lowest category. Multiple estimates from the same study were included in the same meta-analysis where it was clear that samples did not overlap. Where heterogeneity was identified, a sub-group analysis on the effect size by menopausal status at baseline was conducted. Sensitivity analyses were also completed to assess study estimate differences by exogenous hormone use (i.e., oral-contraceptive use or hormonal replacement therapy). Where it was not indicated that hormone use was controlled for either in statistical analysis or by sample restriction, the study was left out of the sensitivity analysis. A one-stage, dose–response meta-analysis of summarized data using restricted cubic splines was performed (17). The Risk of Bias in Nonrandomized Studies-of-Exposures (ROBINS-E; ref. 18) tool was used to evaluate risk of bias (ROB) for the observational studies. The ROBINS-E tool was not developed to assess Mendelian randomization studies. There is currently no validated tool to assess ROB in Mendelian randomization studies. The overall quality of evidence and strength of the findings were assessed using the Grading of Recommendations Assessment, Development and Evaluation (GRADE) system (19). All statistical analyses were performed using Stata version 16 (Stata Corporation, College Station, Texas).

## Results

### Search results

**Figure 1.**
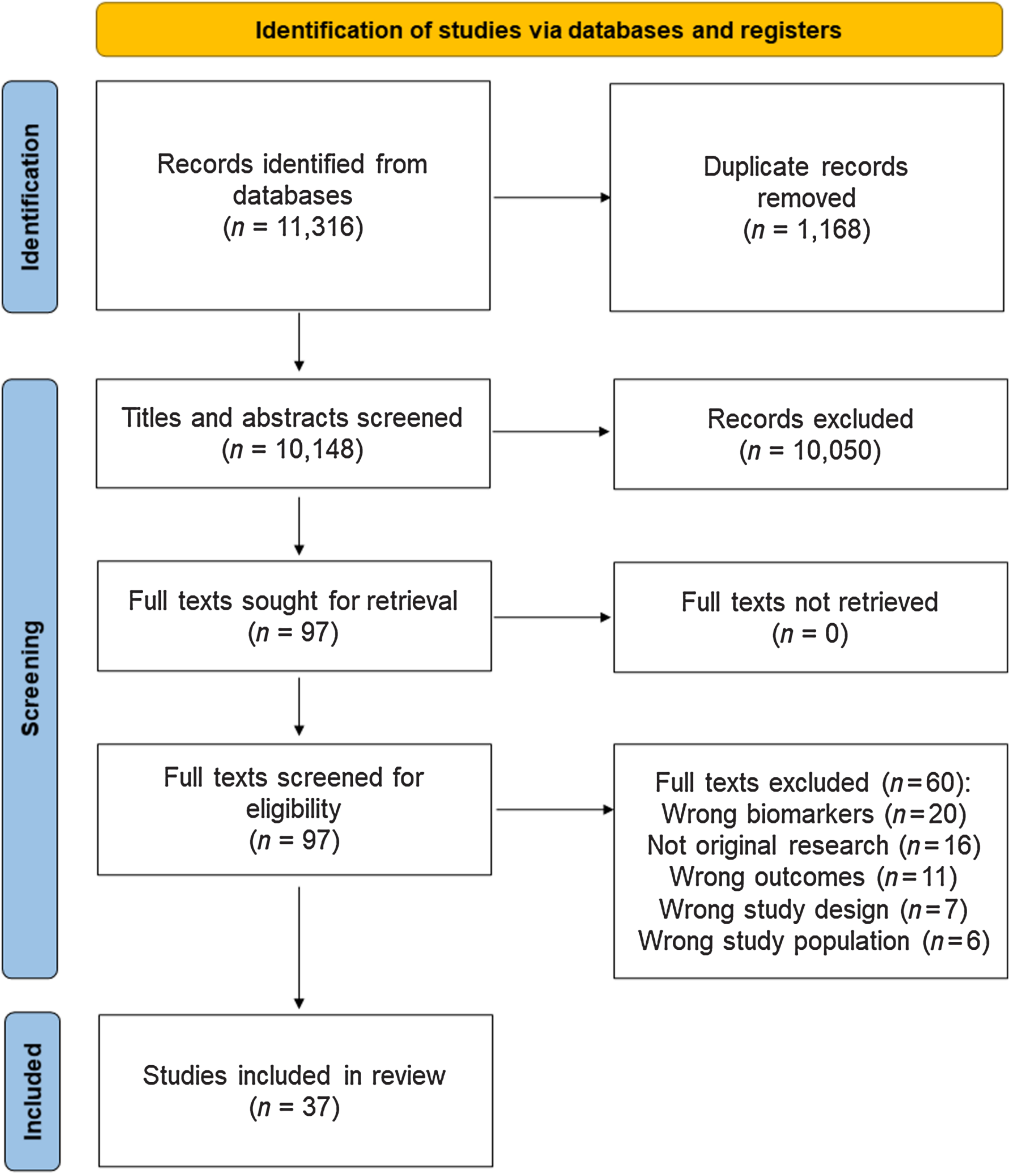
PRISMA flow diagram. This figure incorporates literature search, screening and study selection.

The search results are presented in Fig. 1. Of the 11,316 publications retrieved in the search, 37 investigated the effect of inflammatory biomarkers on breast cancer risk. Of these, 34 publications were observational studies (26 independent cohorts; refs. 11, 20–50, 51, 52), and 3 publications were two-sample Mendelian randomization analyses using summary-level data (53–55). The Mendelian randomization publications used SNPs that predicted a proportion of circulating inflammatory markers in UK Biobank Study (UKBB; ref. 55) participants as instrumental variables for the biomarkers of interest. These genetic proxies were used as the exposures of interest in analyses of breast cancer cases and controls from a large, international consortium (BCAC; refs. 53–55). Both studies predominantly included people of European ancestry.

### Study characteristics

Characteristics of the included studies are detailed in the Supplementary Methods and Materials (Supplementary Tables S2A and S2B). Follow-up time in eligible studies ranged from less than one year to 25 years. All observational studies included postmenopausal women at baseline blood collection (*n* = 26 independent studies; 34 publications), with 16 of these also including premenopausal women (18 publications; refs. 20, 21, 26, 28, 29, 34, 38, 41–43, 46–50, 11, 51, 52). The sample size of studies ranged between 142 to 7,938 for premenopausal women and 302 to 44,715 for postmenopausal women. Inflammatory biomarkers examined in the studies included blood concentrations of CRP (*n* = 17; refs. 11, 20–22, 24, 28–30, 33, 35, 39–42, 44, 46, 48–52), TNFα (*n* = 4; refs. 20, 27, 33, 38), leptin (*n* = 9; refs. 20, 26, 32–34, 40, 43, 45, 47), adiponectin (*n* = 9; refs. 20, 26, 30, 31, 33, 40, 47, 48), IL6 (*n* = 4; refs. 20, 27, 33, 35), IL8 (*n* = 1; ref. 27), IL1β (*n* = 1; ref. 27), CCL2 (*n* = 2; refs. 43, 47) and urinary prostaglandin E_2_ (PGE_2_) metabolites (*n* = 3; refs. 36, 37). The Mendelian randomization studies were performed on two-sample, summary-level data. The sample sizes for the outcome studies were 298,951 (BCAC; refs. 53–55) and 367,643 (UKBB; ref. 55). One Mendelian randomization study was available for each of CRP (54), TNFα (55), adiponectin (54), IL6 (54), IL13 (53), and CCL2 (53).

### ROB

ROB assessments are presented in the Supplementary Methods and Materials (Supplementary Table S3). All observational studies had at least moderate ROB, due to the potential for uncontrolled or residual confounding. Assessment results found one serious (49) and 31 moderate (11, 20–48, 50–52) ROB observational studies. The study with serious ROB did not control for confounding arising from adiposity, alcohol, or diet, and did not provide an explanation for not doing so (49). All other observational studies adjusted for age and body mass index (BMI; or another proxy for adiposity). Diet and/or alcohol were not controlled for in several studies (24–26, 34, 35, 38, 42, 45), with a few indicating that adjustments for these factors did not change risk estimates (29, 31, 39–41, 44). ROB in exposure classification was low for most studies, though it was deemed moderate for studies with high (>15% variation; ref. 56) or missing information on intra- and inter-assay variation (21, 24, 27, 38, 41, 44, 52) and poor sensitivity (limits of detection, biomarker-dependent; refs. 49, 51). The three Mendelian randomization studies had potential bias arising from weak genetic instruments and horizontal pleiotropy, although two studies had employed sensitivity analyses to test results in the presence of potential bias (53, 54).

### Effect of inflammatory biomarkers on breast cancer risk

**Figure 2.**
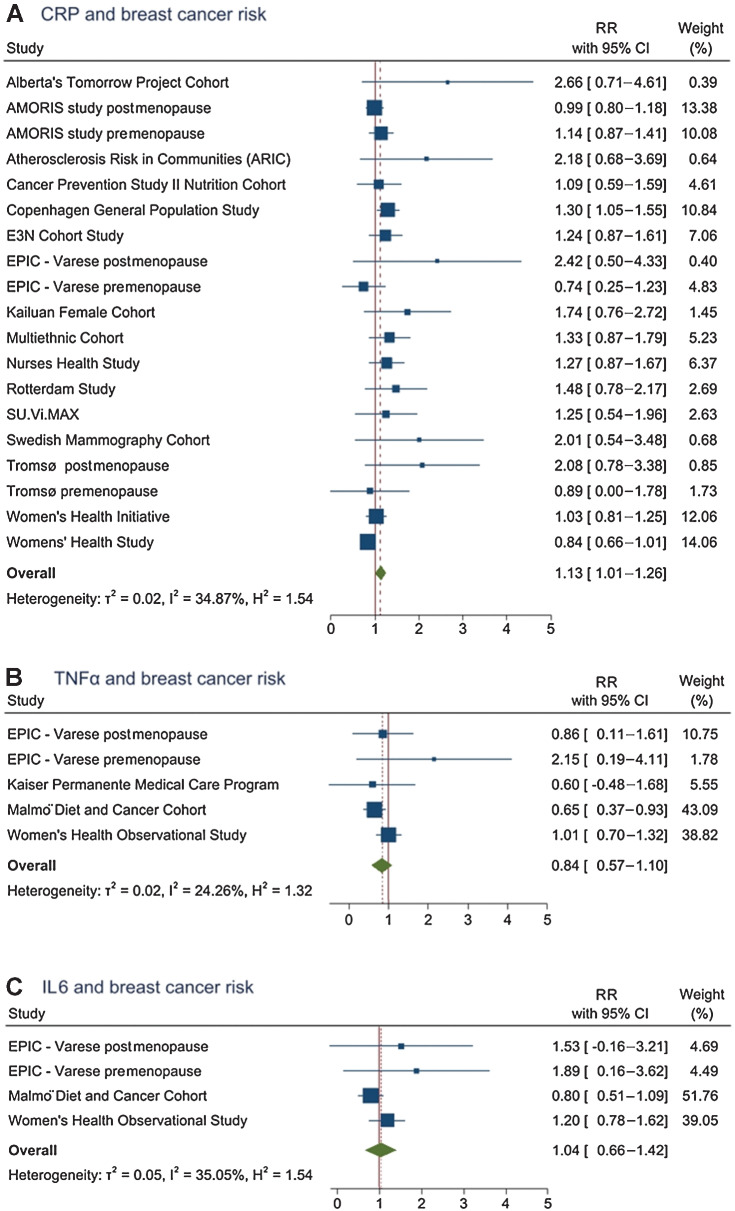
Forest plots for effects of inflammatory biomarkers on breast cancer risk. Forest plots for (**A**) CRP, (**B**) TNFα, and (**C**) IL6.

**Figure 3.**
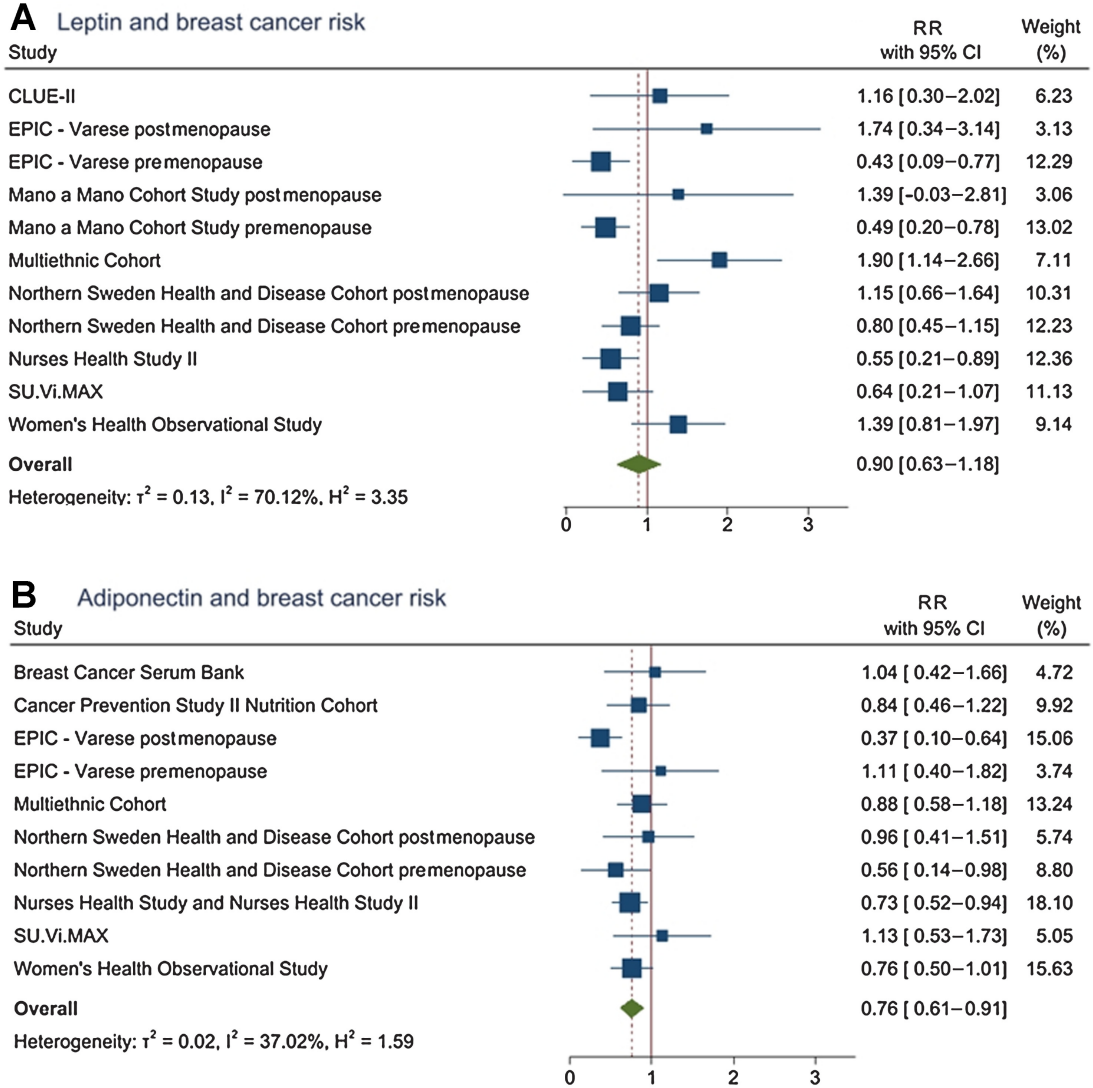
Forest plots for effects of inflammatory biomarkers on breast cancer risk. Forest plots for (**A**) leptin and (**B**) adiponectin.

**Figure 4.**
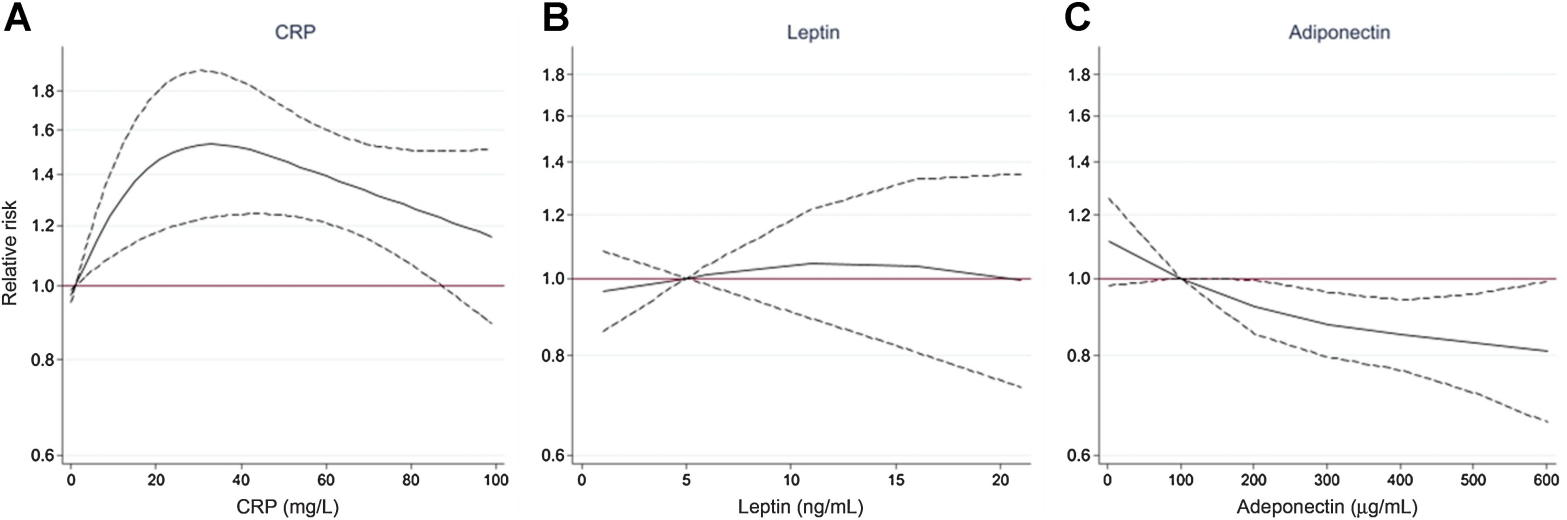
Dose–response curves for effects of inflammatory biomarkers on breast cancer risk. Dose–response curves for (**A**) CRP, (**B**) leptin, and (**C**) adiponectin.

Meta-analysis results for each inflammatory biomarker are presented as forest plots and can be found in Figs. 2 and 3 for overall risk of breast cancer, and Supplementary Methods and Materials (Supplementary Figs. S1–S5) for subgroup and sensitivity analyses. Where categorical data were available, estimates compared the highest category level of the biomarker to the lowest category level. Dose–response curves for overall breast cancer risk are presented in Fig. 4 (CRP, leptin, adiponectin) and by menopausal subgroups in Supplementary Methods and Materials (Supplementary Figs. S1D, S4C, and S5C).

### CRP

CRP was associated with an increased risk of breast cancer for women with the highest levels of CRP compared with the lowest levels [*n* = 16; risk ratio (RR) = 1.13; 95% confidence interval (CI), 1.01–1.26; I^2^ = 34.87%; Fig. 2). The CRP-breast cancer relationship appeared to be dose-dependent, with a potential inverse U relationship (Fig. 4). In a sub-group analysis, there was little evidence of an effect in premenopausal women (*n* = 6; RR = 1.02; 95% CI, 0.84–1.21; I^2^ = 0%; Supplementary Methods and Materials; Fig. 1A). For postmenopausal women, CRP was positively associated with an increased risk of breast cancer for the highest category compared with the lowest (*n* = 14; RR = 1.16; 95% CI, 0.98–1.34; I^2^ = 44.12%), though the estimate was imprecisely estimated (Supplementary Methods and Materials; Fig. 1A). The Mendelian randomization study did not find evidence for an effect of CRP on breast cancer risk (OR per 1-SD increase = 1.03; 95% CI, 0.94–1.13; *P* = 0.48); no difference was reported between ER+ and ER- breast cancers (54). Of the 15 studies that measured CRP, 12 used measures for high-sensitivity CRP (hsCRP) or had a lower limit of detection of at least 0.3 mg/L (11, 20–22, 24, 28, 29, 41, 44, 46, 47, 50). Sensitivity analyses excluding studies with unknown status for exogenous hormone use (Supplementary Methods and Materials; Fig. 1B), or studies with high ROB (Supplementary Methods and Materials; Fig. 1C), did not substantially change the RR or heterogeneity estimate.

### TNF**α**

Meta-analysis suggested that the highest category of TNFα compared with the lowest category was associated with a possible decreased risk of breast cancer (*n* = 4; RR = 0.84; 95% CI, 0.57–1.10; I^2^ = 24.3%; Fig. 2). Subgroup analysis could not explain the heterogeneity, as this remained high for each subgroup including pre- (I^2^ = 70%) and postmenopausal women (I^2^ = 89%; Supplementary Methods and Materials; Fig. 2A). Sensitivity analyses excluding studies with unknown status for exogenous hormone use (Supplementary Methods and Materials; Fig. 2B) did not substantially change the risk estimate or heterogeneity estimate. Consistent with the meta-analysis, a Mendelian randomization study found strong evidence that TNFα decreased breast cancer risk (OR per 1-SD increase = 0.51; 95% CI, 0.39–0.67; ref. 55).

### Interleukins

The only interleukin with sufficient studies (*n* = 3) to conduct a meta-analysis was IL6. We found little evidence of an association with breast cancer risk (RR = 1.04; 95% CI, 0.66–1.42; I^2^ = 35.05%; Fig. 2). There was insufficient evidence to determine any differences by menopause subgroup (Supplementary Methods and Materials; Fig. 3A). A Mendelian randomization study found some evidence of a positive causal effect (OR per 1-SD increase = 1.09; 95% CI, 0.96–1.25; ref. 54).

In an individual Mendelian randomization study, higher levels of IL13 led to a small increase in breast cancer risk in another (OR per 1-SD increase = 1.06; 95% CI, 1.03–1.10; ref. 53). In a nested case–control study, there was a small increase in postmenopausal breast cancer risk was noted for increasing levels of IL8 (OR highest versus lowest category = 1.09; 95% CI, 0.71–1.66), though estimates were imprecisely estimated as confidence intervals (CI) were wide (27).

### Leptin

Following meta-analysis, women with the highest levels of leptin were not clearly more likely to develop breast cancer compared with women with the lowest levels (*n* = 8; RR = 0.90; 95% CI, 0.63–1.18; I^2^ = 70.1%; Fig. 3). There was no evidence of a dose–response relationship (Fig. 4).

Subgroup analysis suggested a possible difference in breast cancer risk for pre- and postmenopausal levels of circulating leptin. For premenopausal women, a meta-analysis found that those with the highest levels compared with the lowest had decreased breast cancer risk (*n* = 4; RR = 0.56; 95% CI, 0.39–0.72; I^2^ = 0.0%; Supplementary Methods and Materials; Fig. 4A). Conversely, for postmenopausal women, higher levels of leptin may have been associated with increased breast cancer risk (*n* = 7; RR = 1.23; 95% CI, 0.86–1.60; I^2^ = 46.8%). However, there was moderate heterogeneity and a wide CI, limiting the certainty of this result. In a sensitivity analysis excluding studies with unknown status for exogenous hormone use, the positive association in postmenopausal women was strengthened, with a large reduction in heterogeneity (*n* = 5; RR = 1.37; 95% CI, 1.06–1.67; I^2^ = 0.0%; Supplementary Methods and Materials; Fig. 4B).

### Adiponectin

Women with the highest levels of adiponectin experienced a decreased risk in overall breast cancer risk (*n* = 8; RR = 0.76; 95% CI, 0.61–0.91; I^2^ = 37.02%), compared with those with the lowest levels (Fig. 3). A sensitivity analysis excluding studies with unknown status for exogenous hormone use minimally changed the risk estimate (*n* = 7; RR = 0.72; 95% CI, 0.56–0.87; I^2^ = 38.4%; Supplementary Methods and Materials; Fig. 5B). Sub-group analyses revealed that adiponectin is protective for postmenopausal women (*n* = 3; RR = 0.75; 95% CI, 0.57–0.92; I^2^ = 47.0%; Supplementary Methods and Materials; Fig. 5A), and showed evidence of a dose–response effect (Supplementary Methods and Materials; Fig. 5C). There was little evidence of an association for premenopausal women, with high heterogeneity (RR = 0.93; 95% CI, 0.44–1.41; I^2^ = 52.4%). A Mendelian randomization study did not support a causal effect of adiponectin on breast cancer risk (OR per 1-SD increase = 1.06; 95% CI, 0.81–1.40), with wide and overlapping CIs from the meta-analysis (54).

### Prostaglandins

Three studies examined prostaglandins and breast cancer risk. Urinary metabolite of PGE_2_ was positively associated with postmenopausal breast cancer (HR for highest versus lowest category = 2.01; 95% CI, 1.01–4.29) in one study (36). A second study observed similar results with a dose–response relationship only for the subgroup with BMI < 25 (25). Urinary prostaglandin F_2_-α metabolite was not associated with breast cancer risk in a third study (23).

### Other cytokines

In individual studies, there was contrasting evidence of an effect of CCL2. Observational studies did not clearly support an increased risk for higher compared with lower levels of CCL2 and breast cancer risk; (OR = 1.14; 95% CI, 0.70–1.85; ref. 47) and (OR = 1.01; 95% CI, 0.48–1.74; ref. 43). In contrast, in a Mendelian randomization study genetically predicted circulating levels of CCL2 increased overall breast cancer risk (OR per 1-SD increase = 1.08; 95% CI, 1.03–1.12; ref. 53).

## GRADE

Table 1 presents results of the GRADE appraisal for biomarkers where there were sufficient studies to conduct a meta-analysis. Initially, the evidence for all inflammatory biomarkers and breast cancer risk was graded as low, as the results were based on observational studies only. The quality of evidence for CRP and breast cancer risk was upgraded to moderate due to evidence of a dose–response relationship. Evidence for TNFα and breast cancer risk was also upgraded as moderate due to the triangulation of evidence, with consistency in direction of effect between observational and Mendelian randomization studies. The quality of evidence for IL6 was downgraded to very low due to imprecision and inconsistency in the available evidence of only 3 studies. The quality of evidence for adiponectin was graded up due to a large magnitude of effect.

**Table 1.** GRADE appraisal for inflammatory biomarkers – breast cancer pathways.

Inflammatory biomarker	Study type, number, sample size	Meta-analysis effect estimates (RR, 95% CI)	Dose–response	Quality of evidence
**CRP**				
Overall	Observational, 16 (153,669)	1.13 (1.01–1.26)	Inverted U	
	Mendelian randomization, 1 (228,951)	1.03 (0.94–1.13)		Moderate^a^
**TNFα**				
Overall	Observational, 4 (2,256)	0.84 (0.57–1.10)	N/A	
	Mendelian randomization, 1 (596,594)	0.51 (0.39–0.67)		Moderate^b^
**IL6**				
Overall	Observational, 3 (2,152)	1.04 (0.66–1.42)	N/A	
	Mendelian randomization, 1 (228,951)	1.09 (0.96–1.25)		Very low^c^
**Leptin**				
Overall	Observational, 8 (4,291)	0.90 (0.63–1.18)	None	Low
**Adiponectin**				
Overall	Observational, 8 (4,763)	0.76 (0.61–0.91)	Linear	
	Mendelian randomization, 1 (228,951)	1.06 (0.81–1.40)		Moderate^d^

RRs summarize effects comparing the highest versus lowest category of blood biomarker concentration. Mendelian randomization effect estimate represents the OR per standard deviation increase in the exposure.

^a^Graded up due to dose–response effect in observational studies (Fig. 4A).

^b^Graded up due to triangulation of evidence.

^c^Graded down due to imprecision and inconsistency in the available evidence.

^d^Graded up due to substantial magnitude of effect.

## Discussion

In our review of inflammation and breast cancer risk, only increases in CRP were shown to increase breast cancer risk. A possible decrease in breast cancer risk was seen for higher levels of TNFα, leptin and adiponectin; however, wide CIs and heterogeneity limited the certainty of these findings. There was no clear evidence of a relationship between IL6 and breast cancer risk. Evidence was graded as very low (IL6), low (CRP, TNFα, leptin), or moderate (adiponectin).

The robust methodology used to search, synthesize, and appraise the current evidence on inflammatory biomarkers and breast cancer risk is a key strength of our review. The prespecified list of biomarkers in our search strategy improved identification of relevant articles compared with a previous review (7). We have identified five additional studies to the most recent meta-analysis of CRP and breast cancer risk (8), and additional previously unreviewed studies on cytokines (41, 43). Furthermore, only prospective studies and Mendelian randomization studies were included. Given that these study designs have their own strengths and limitations, incorporating findings from both enabled us to triangulate evidence (where risk estimates which align in direction may indicate stronger evidence of a true effect; ref. 57). Unlike observational studies that are inevitably subject to residual confounding, Mendelian randomization studies harness the randomization of genetic assignment at conception to measure causal effects between genetic proxies and the outcome, akin to a trial. In our review, triangulation of evidence was observed for TNFα, where higher levels were associated with decreased breast cancer risk in both a meta-analysis of observational studies and a Mendelian randomization study.

This systematic review revealed a sparsity of evidence from prospective cohorts and Mendelian randomization studies. Of the 13 biomarkers searched, publications included in the review examined 10, and only five biomarkers met criteria to facilitate a meta-analysis. Studies that measured biomarkers in premenopausal women were limited (17 of the 34 publications). Given that risk factors and mechanisms for breast cancer development likely differ between pre- and postmenopausal women (2, 58), and that ovulation itself appears to parallel with inflammation (59), more evidence is required to further clarify mechanisms in premenopausal women.

There was moderate to high heterogeneity noted for all meta-analyses. Subgroup meta-analyses were conducted to assess any potential differences by menopausal status. While menopause explained some of the heterogeneity for leptin, the sources of heterogeneity could not be further clarified for other markers due to the limited reporting of stratified results and inconsistent control for confounders between studies.

Risk factors contributing to breast cancer may differ by disease subtypes (60). However, few studies reported results by hormone receptor (HR) status and/or molecular subgroups. Of the studies that did investigate results by HR status, no significant difference was found in most observational studies (26, 33, 34, 52). One study found CRP to be associated with HR-negative breast cancers only (46). Mendelian randomization studies found CCL2 to be associated with overall and HR+ breast cancer (53), IL13 with both HR+ and HR- (53), and no significant differences for CRP, TNFα, adiponectin, and IL6 (54, 55).

Exogenous hormone use was mostly well described in studies and was adjusted for in statistical analyses, as is typical in studies on breast cancer. We included women who were taking exogenous hormones in our main analyses, although sensitivity analyses removed these and findings remained consistent. In contrast, the use of NSAIDs or aspirin was not routinely reported or adjusted for. This may be because NSAID and aspirin use is more difficult to measure, and use is typically occasional. Anti-inflammatory medication may pose a potential source of confounding in the prospective cohort studies. A recent meta-analysis of randomized controlled trials suggested that use of low-dose aspirin may offer a small degree of protection against breast cancer (61).

Almost all studies measured biomarkers only once at baseline. Correspondingly, exposure measurements were potentially limited by non-differential misclassification, leading to a potential underestimation of effects. Sensitivity analyses excluding cases diagnosed within 12/24 months of blood draw were not consistently reported amongst studies. However, no significant differences in risk estimates were observed in studies that conducted sensitivity analyses investigate risk with a time-lag until diagnosis (51, 52). Funnel plots of the included studies indicated that smaller studies with null or weak associations may be missing for CRP, leptin, and adiponectin.

Prior narrative reviews suggest that inflammation increases cancer risk (3, 62). Chronic systemic inflammation may increase breast cancer risk by promoting continuous tissue remodeling and creating a tumorigenic environment in breast tissue (6, 63). For example, CRP is an acute-phase protein produced in the liver in response to pro-inflammatory signals from cytokines such as TNFα and IL6, and moderately high CRP is indicative of low-grade inflammation (64). Higher adiponectin is observed in more active people and those with lower adiposity, and is typically associated with decreased inflammation (65, 66). While our meta-analysis of prospective cohort studies supported the protective role of increased adiponectin, results from the Mendelian randomization study did not support a causal effect of adiponectin on breast cancer risk.

The results for TNFα were not anticipated, as increased levels of the pro-inflammatory biomarker appeared to decrease risk of breast cancer. These findings should be interpreted with caution due to the small number of studies (*n* = 4), and it may reflect the complex dual roles of TNFα in both its antitumor functions and inflammatory tumor-promoting functions (67). Results for leptin were also not expected as it is hypothesized to promote breast cancer through increasing circulating hormone levels via aromatization of androgens to estrogens (65). Leptin was associated with increased risk in postmenopausal women but decreased risk in premenopausal women. Interestingly, these effects were analogous to the associations between adiposity and pre- and postmenopausal breast cancer (2). While all studies examining leptin controlled for BMI, it is possible that residual confounding due to inadequate control for adiposity could explain the observed relationship.

The overarching aim of this two-part series of reviews was to determine whether the inflammation mediates the inverse relationship between physical activity and breast cancer risk. Although Part 1 identified decreases in CRP, TNFα, IL6, and leptin following exercise interventions (8), in Part 2 (this manuscript) only CRP was associated with increased breast cancer risk. While both the quality of evidence identified as well as the complex nature of inflammation should caution against simple conclusions, overall, these two reviews do not provide clear evidence for a physical activity – inflammation – breast cancer pathway. This does not eliminate the possibility of this pathway; however better-quality, large-scale studies are required clarify the evidence. Future prospective studies should investigate a wider range of biomarkers of inflammation, obtaining samples from both pre- and postmenopausal women.

## Supplementary Material

Figure S1A Supplementary Figure 1A presents forest plots for CRP and breast cancer risk, by menopausal status

Figure S1B Supplementary Figure 1B presents forest plots for CRP and breast cancer risk, excluding studies where exogenous hormone use status was unknown

Figure S1C Supplementary Figure 1C present forest plots for CRP and breast cancer risk, excluding studies that had a moderate risk of bias

Figure S1D Supplementary Figure 1D presents dose-response curves for CRP and breast cancer risk, by menopausal status

Figure S1E Supplementary Figure 1E presents the funnel plot from the meta-analysis of CRP and breast cancer risk

Figure S2A Supplementary Figure 2A presents forest plots for TNF-alpha and breast cancer risk, by menopausal status

Figure S2B Supplementary Figure 2B presents forest plots for TNF-alpha and breast cancer risk, excluding studies where exogenous hormone use status was unknown

Figure S2C Supplementary Figure 2C presents the funnel plot from the meta-analysis of TNF-alpha and breast cancer risk

Figure S3A Supplementary Figure 3A presents forest plots for IL-6 and breast cancer risk, by menopausal status

Figure S3B Supplementary Figure 3B presents the funnel plot from the meta-analysis of IL-6 and breast cancer risk

Figure S4A Supplementary Figure 4A presents forest plots for leptin and breast cancer risk, by menopausal status

Figure S4B Supplementary Figure 4B presents forest plots for leptin and breast cancer risk, excluding studies where exogenous hormone use status was unknown

Figure S4C Supplementary Figure 4C presents dose-response curves for leptin and breast cancer risk, by menopausal status

Figure S4D Supplementary Figure 4D presents the funnel plots from the meta-analysis of leptin and breast cancer risk

Figure S5A Supplementary Figure 5A presents the forest plots for adiponectin and breast cancer risk, by menopausal status

Figure S5B Supplementary Figure 5B presents forest plots for adiponectin and breast cancer risk, excluding studies where exogenous hormone use status was unknown

Figure S5C Supplementary Figure 5C presents dose-response curves for adiponectin and breast cancer risk, by menopausal status

Figure S5D Supplementary Figure 5D presents the funnel plots from the meta-analysis of adiponectin and breast cancer risk

Table S1 Supplementary Table 1 presents the search terminology for the systematic review

Table S2A Supplementary Table 2A presents the study characteristics of the Mendelian randomization studies

Table S2B Supplementary Table 2B presents the study characteristics of the prospective cohort studies

Table S3 Supplementary Table 3 presents the risk of bias assessment, using the ROBINS-E
