# Supplementary material for: Linking Physical Activity to Breast Cancer via Inflammation, Part 2: The Effect of Inflammation on Breast Cancer Risk
Source: Cancer Epidemiol Biomarkers Prev. 2023 Mar 3;32(5):597–605. doi: 10.1158/1055-9965.EPI-22-0929 (PMC10150245; doi:10.1158/1055-9965.EPI-22-0929)
Supplement: Figure S1C — Supplementary Figure 1C present forest plots for CRP and breast cancer risk, excluding studies that had a moderate risk of bias [file epi-22-0929_figure_s1c_suppsf1c.docx]

**Supplementary Figure 1C: Forest plot of CRP and breast cancer risk estimates, sensitivity analysis excluding studies with moderate risk of bias on exposure classification**

**
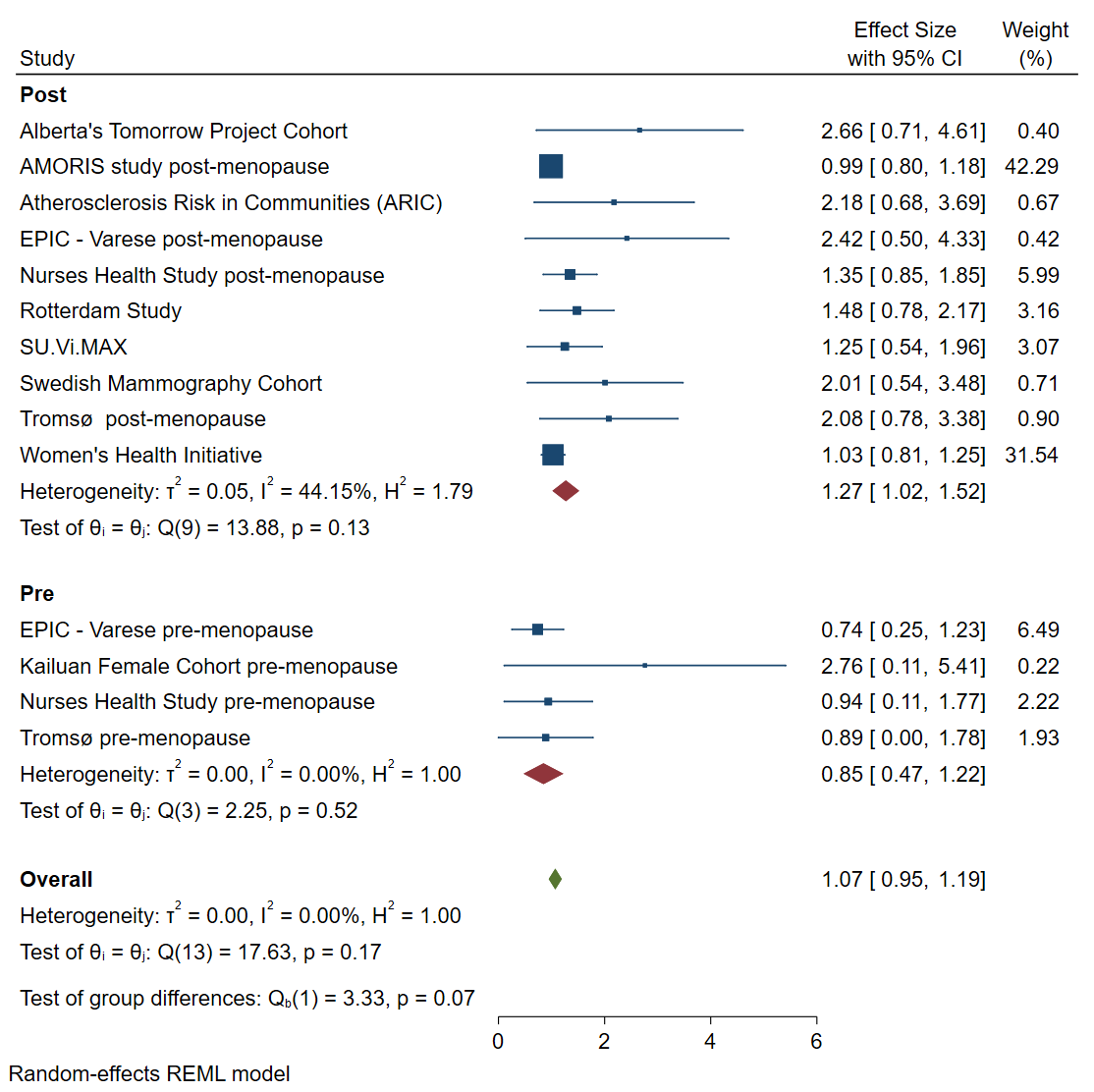
**
