# Supplementary figures and images for: Linking Physical Activity to Breast Cancer via Inflammation, Part 2: The Effect of Inflammation on Breast Cancer Risk
Source: Cancer Epidemiol Biomarkers Prev. 2023 Mar 3;32(5):597–605. doi: 10.1158/1055-9965.EPI-22-0929 (PMC10150245; doi:10.1158/1055-9965.EPI-22-0929)

**Supplementary Figure 1D: CRP dose-response curves by menopause subgroup**

**
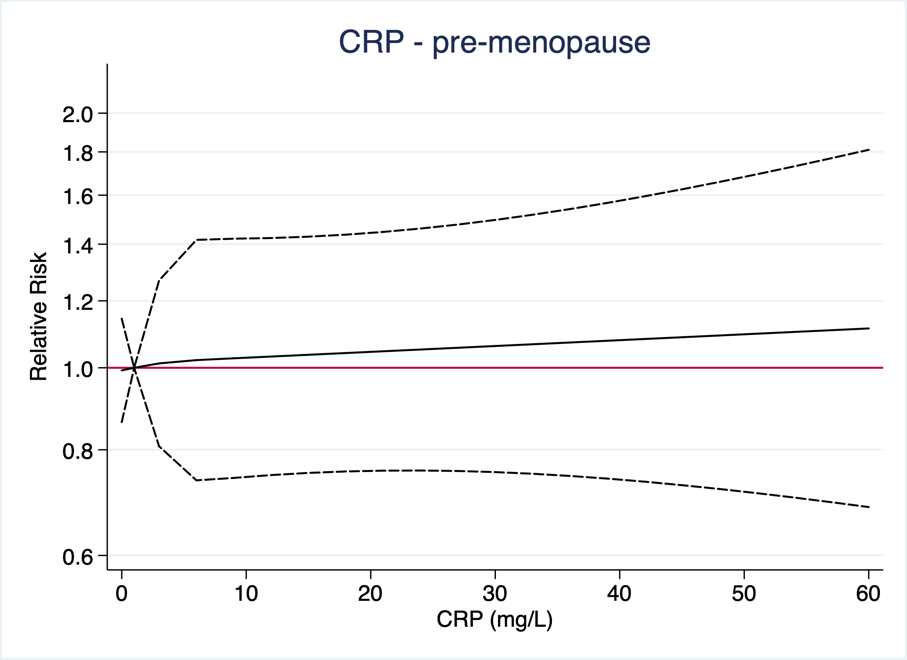
**

**
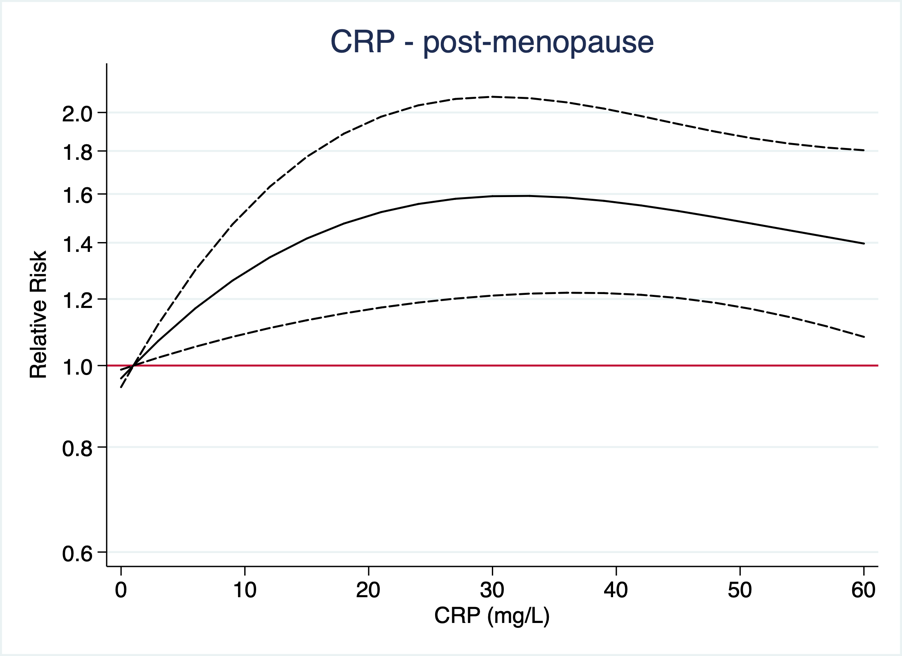
**

Supplement: Figure S1D — Supplementary Figure 1D presents dose-response curves for CRP and breast cancer risk, by menopausal status [file epi-22-0929_figure_s1d_suppsf1d.docx]

**Supplementary Figure 1E: CRP and breast cancer meta-analysis funnel plot**

**
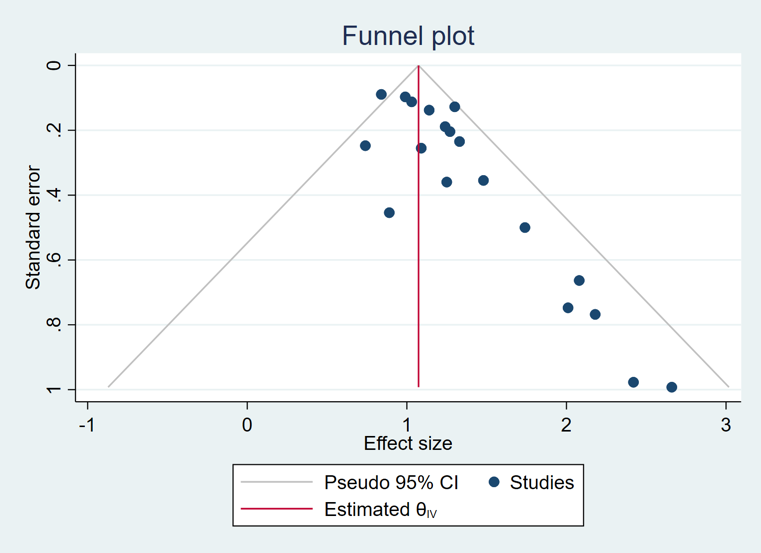
**

Supplement: Figure S1E — Supplementary Figure 1E presents the funnel plot from the meta-analysis of CRP and breast cancer risk [file epi-22-0929_figure_s1e_suppsf1e.docx]

**Supplementary Figure 2C: TNF-α and breast cancer meta-analysis funnel plot**

**
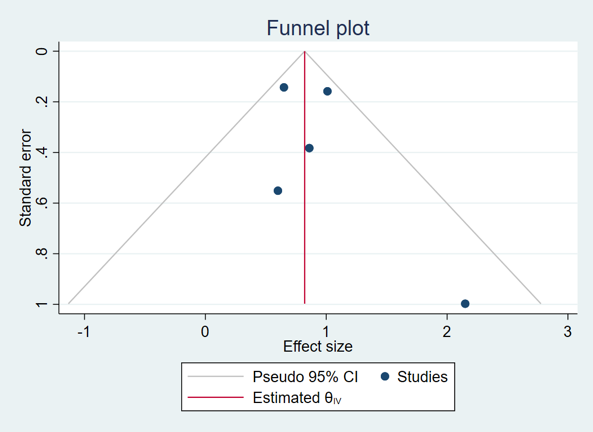
**

Supplement: Figure S2C — Supplementary Figure 2C presents the funnel plot from the meta-analysis of TNF-alpha and breast cancer risk [file epi-22-0929_figure_s2c_suppsf2c.docx]

**Supplementary Figure 3B: IL-6 and breast cancer meta-analysis funnel plot**

**
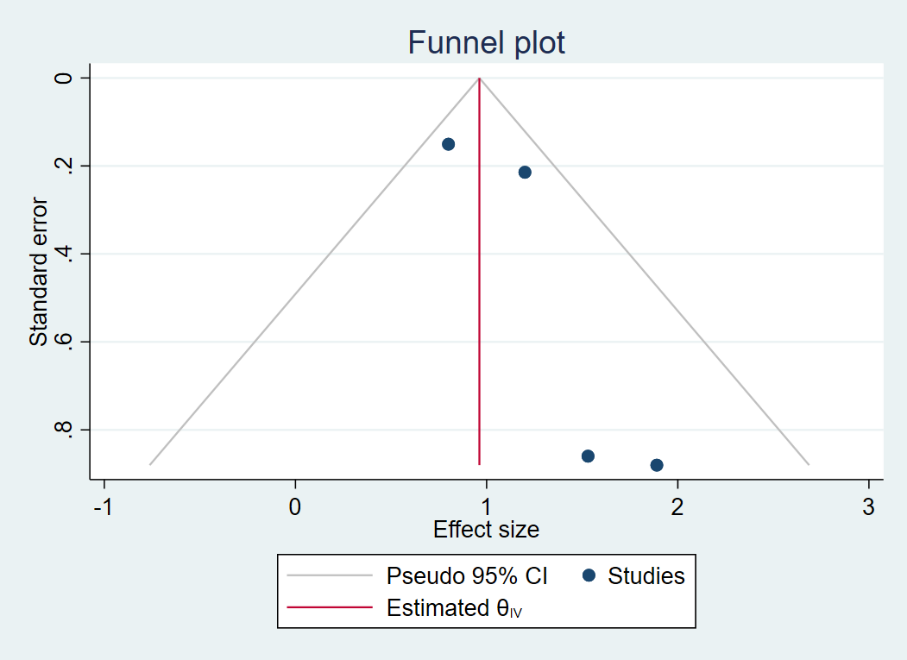
**

Supplement: Figure S3B — Supplementary Figure 3B presents the funnel plot from the meta-analysis of IL-6 and breast cancer risk [file epi-22-0929_figure_s3b_suppsf3b.docx]

**Supplementary Figure 4C: Leptin dose-response curves by menopause subgroup**

**
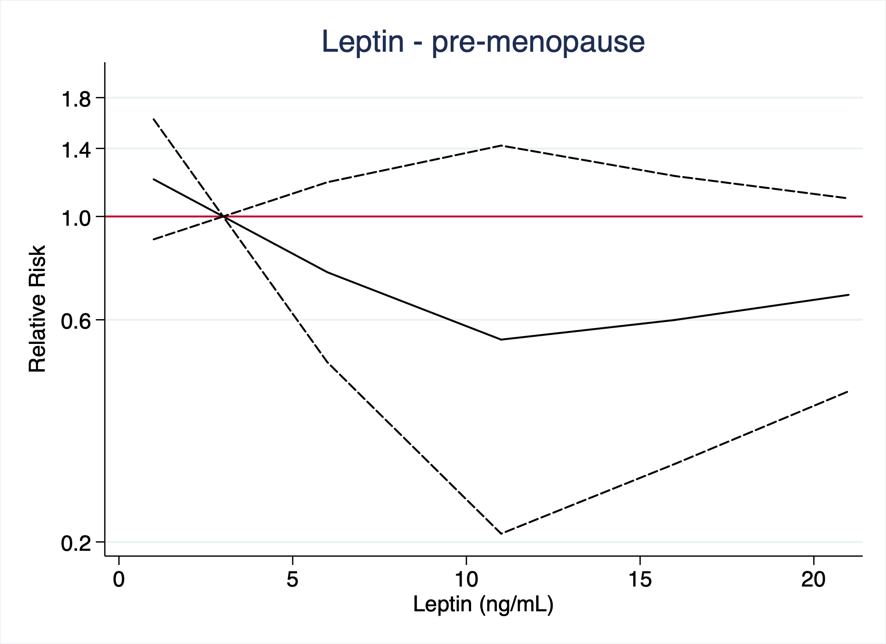
**

**
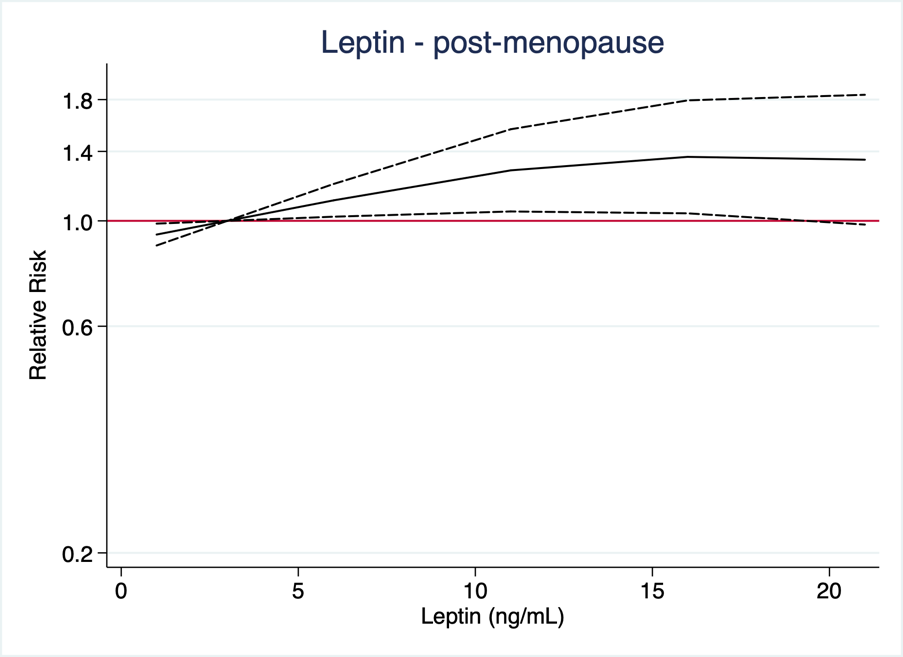
**

Supplement: Figure S4C — Supplementary Figure 4C presents dose-response curves for leptin and breast cancer risk, by menopausal status [file epi-22-0929_figure_s4c_suppsf4c.docx]

**Supplementary Figure 4D: Leptin and breast cancer risk meta-analysis funnel plot**

**
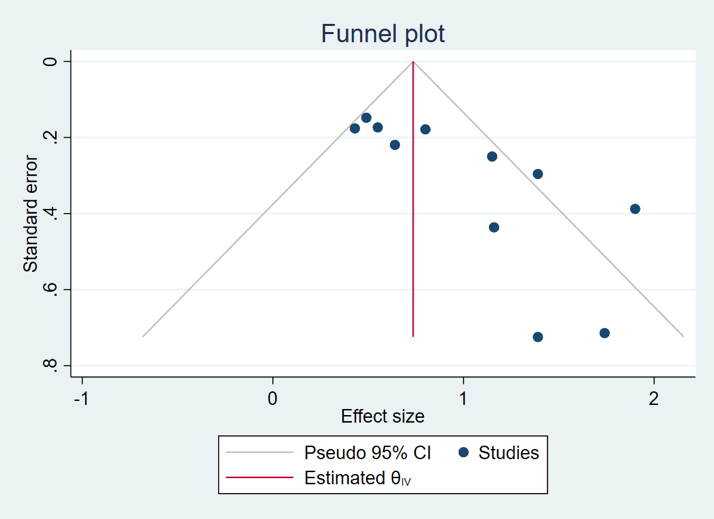
**

Supplement: Figure S4D — Supplementary Figure 4D presents the funnel plots from the meta-analysis of leptin and breast cancer risk [file epi-22-0929_figure_s4d_suppsf4d.docx]

**Supplementary Figure 5C: Adiponectin dose-response curves by menopause subgroup**

**
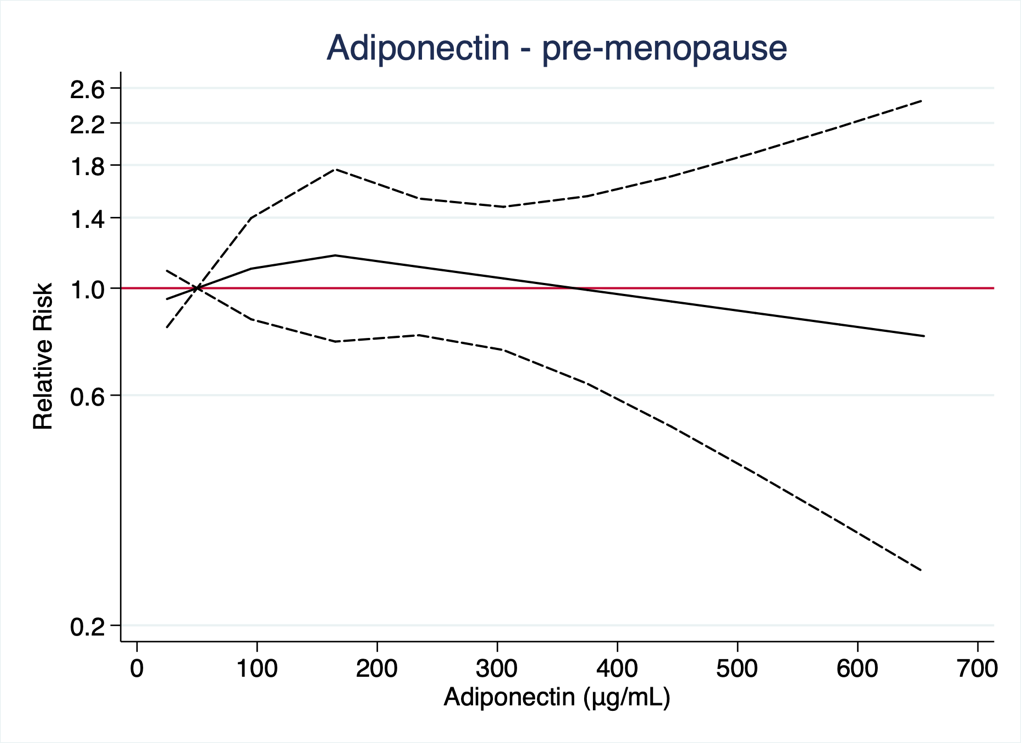
**

**
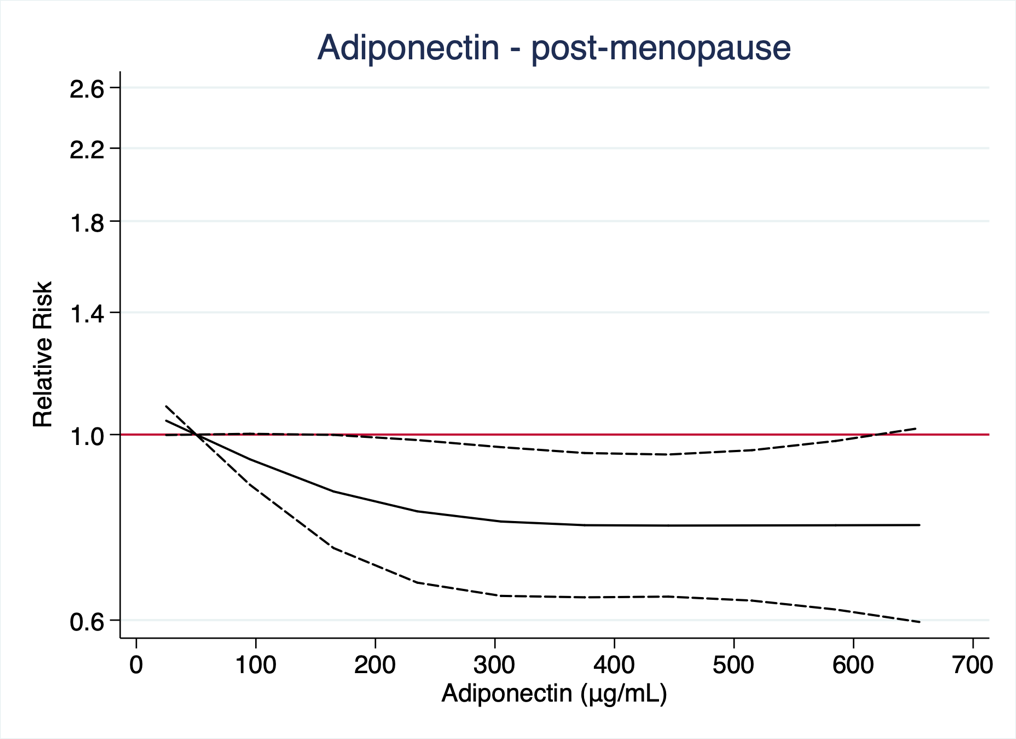
**

Supplement: Figure S5C — Supplementary Figure 5C presents dose-response curves for adiponectin and breast cancer risk, by menopausal status [file epi-22-0929_figure_s5c_suppsf5c.docx]

**Supplementary Figure 5D: Adiponectin and breast cancer risk meta-analysis funnel plot**

**
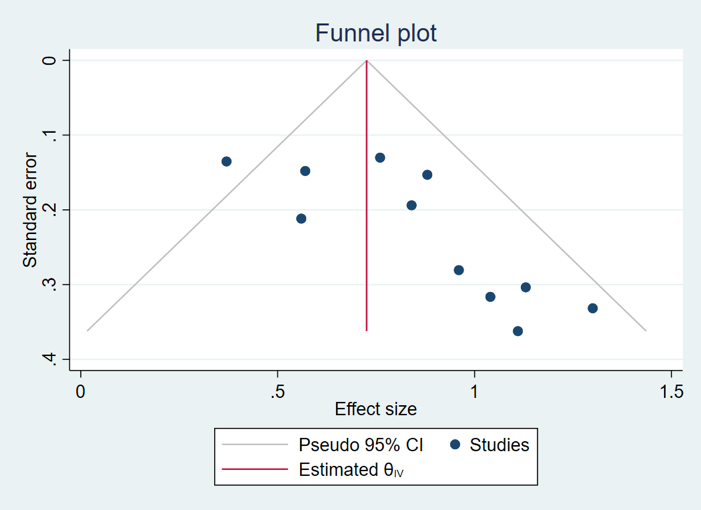
**

Supplement: Figure S5D — Supplementary Figure 5D presents the funnel plots from the meta-analysis of adiponectin and breast cancer risk [file epi-22-0929_figure_s5d_suppsf5d.docx]
