# Supplementary material for: Linking Physical Activity to Breast Cancer via Inflammation, Part 2: The Effect of Inflammation on Breast Cancer Risk
Source: Cancer Epidemiol Biomarkers Prev. 2023 Mar 3;32(5):597–605. doi: 10.1158/1055-9965.EPI-22-0929 (PMC10150245; doi:10.1158/1055-9965.EPI-22-0929)
Supplement: Figure S3A — Supplementary Figure 3A presents forest plots for IL-6 and breast cancer risk, by menopausal status [file epi-22-0929_figure_s3a_suppsf3a.docx]

**Supplementary Figure 3A: Forest plot of IL-6 and breast cancer risk estimates, by menopause subgroup**

**
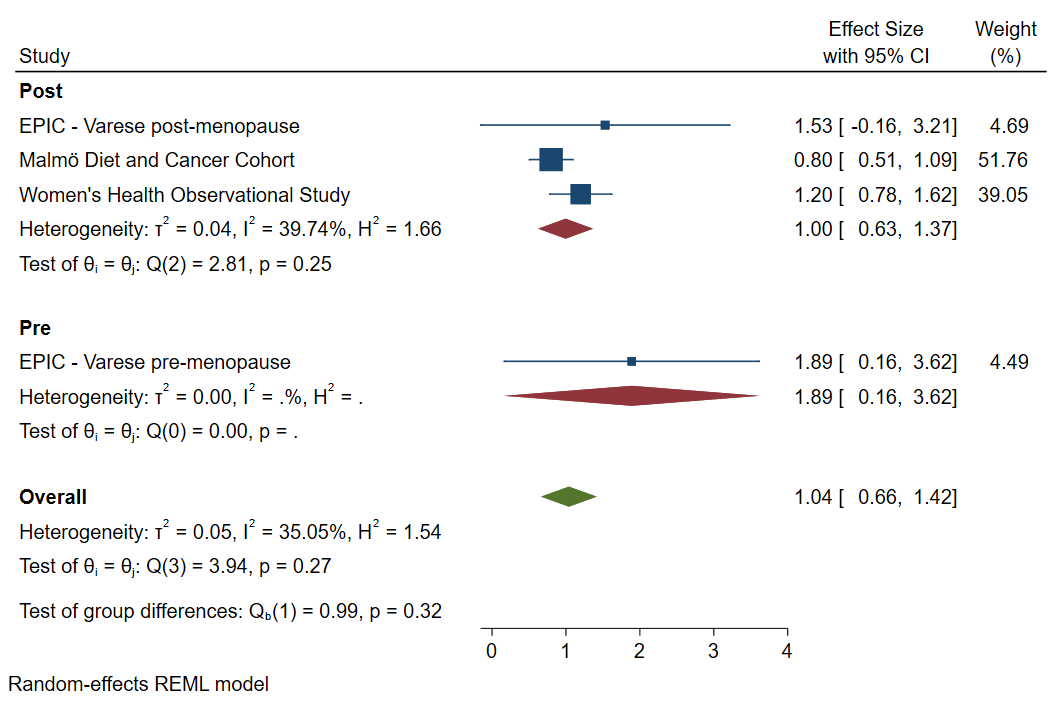
**
