# Supplementary material for: Linking Physical Activity to Breast Cancer via Inflammation, Part 2: The Effect of Inflammation on Breast Cancer Risk
Source: Cancer Epidemiol Biomarkers Prev. 2023 Mar 3;32(5):597–605. doi: 10.1158/1055-9965.EPI-22-0929 (PMC10150245; doi:10.1158/1055-9965.EPI-22-0929)
Supplement: Figure S4B — Supplementary Figure 4B presents forest plots for leptin and breast cancer risk, excluding studies where exogenous hormone use status was unknown [file epi-22-0929_figure_s4b_suppsf4b.docx]

**Supplementary Figure 4B: Forest plot of leptin and breast cancer risk estimates, sensitivity analysis excluding studies with unknown status for exogenous hormone use**

**
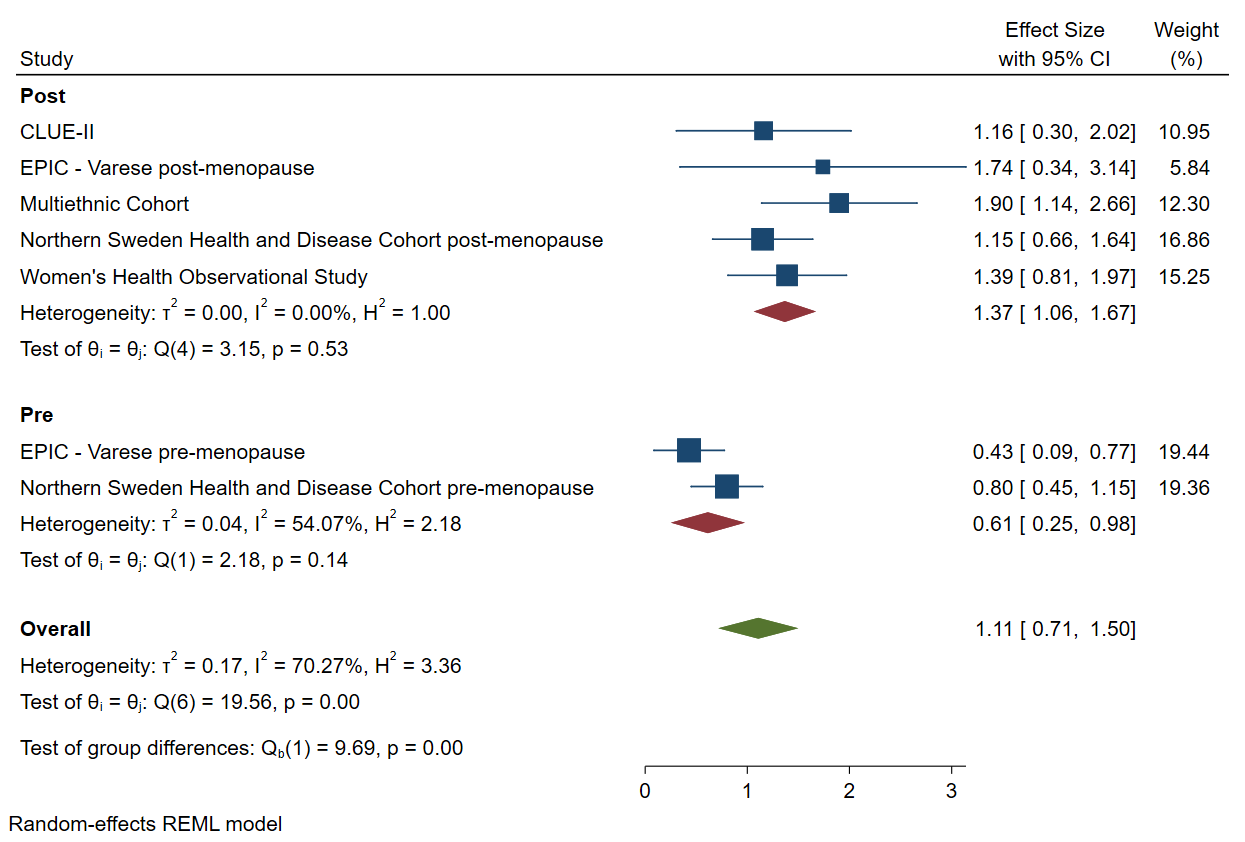
**
