# Supplementary material for: Linking Physical Activity to Breast Cancer via Inflammation, Part 2: The Effect of Inflammation on Breast Cancer Risk
Source: Cancer Epidemiol Biomarkers Prev. 2023 Mar 3;32(5):597–605. doi: 10.1158/1055-9965.EPI-22-0929 (PMC10150245; doi:10.1158/1055-9965.EPI-22-0929)
Supplement: Table S1 — Supplementary Table 1 presents the search terminology for the systematic review [file epi-22-0929_table_s1_suppst1.docx]

Supplementary Table 1. Search terminology for literature review

| **#** | **Biomarkers** | **Search terms** |
| --- | --- | --- |
| 1 | TNF alpha | Tumor Necrosis Factor-alpha, Cachectin, TNF Superfamily member 2, TNFalpha, TNF-alpha, TNF |
| 2 | Interleukin-6 | Interleukin 6, IL6, B-Cell Stimulatory Factor 2, B-Cell Stimulatory Factor-2, Differentiation Factor-2, B-Cell, Differentiation Factor 2, B Cell, B-Cell Differentiation Factor-2, B Cell Differentiation Factor 2, BSF-2, Hybridoma Growth Factor, Growth Factor, Hybridoma, IFN-beta 2, Plasmacytoma Growth Factor, Growth Factor, Plasmacytoma, Hepatocyte-Stimulating Factor, MGI-2, Myeloid Differentiation-Inducing Protein, B-Cell Differentiation Factor, IL-6, Interferon beta-2, Interferon beta 2, beta-2, Interferon, B Cell Stimulatory Factor-2, B Cell Stimulatory Factor 2 |
| 3 | Leptin | Leptin, Obese Protein, Obese Gene Product, Gene Product, Obese, Ob Gene Product, Gene Product, Ob, Ob Protein |
| 4 | Interleukin-8 | Interleukin 8, IL8, Monocyte-Derived Neutrophil Chemotactic Factor, Neutrophil Activation Factor, Neutrophil-Activating Peptide, Lymphocyte-Derived, Lymphocyte-Derived Neutrophil-Activating Peptide, Neutrophil Activating Peptide, Lymphocyte Derived, Neutrophil-Activating Peptide, Monocyte-Derived, Monocyte-Derived Neutrophil-Activating Peptide, Neutrophil Activating Peptide, Monocyte Derived, Alveolar Macrophage Chemotactic Factor-I, Alveolar Macrophage Chemotactic Factor I, AMCF-I, Anionic Neutrophil-Activating Peptide, Anionic Neutrophil Activating Peptide, Neutrophil-Activating Peptide, Anionic, Peptide, Anionic Neutrophil-Activating, Chemokine CXCL8, CXCL8, Chemokine, Chemokines, CXCL8, CXCL8 Chemokines, Chemotactic Factor, Macrophage-Derived, Chemotactic Factor, Macrophage Derived, Macrophage-Derived Chemotactic Factor, Chemotactic Factor, Neutrophil, Neutrophil Chemotactic Factor, Chemotactic Factor, Neutrophil, Monocyte-Derived, CXCL8 Chemokine, Chemokine, CXCL8, Granulocyte Chemotactic Peptide-Interleukin-8, Chemotactic Peptide-Interleukin-8, Granulocyte, Granulocyte Chemotactic Peptide Interleukin 8, IL-8 |
| 5 | C-Reactive Protein | C-reactive protein, CRP, C reactive protein |
| 6 | Interleukin-10 | Interleukin 10, IL10, IL-10, CSIF-10, Cytokine Synthesis Inhibitory Factor, |
| 7 | Interleukin-1 | Interleukin 1, IL-1, T Helper Factor, Lymphocyte-Activating Factor, Lymphocyte Activating Factor, Macrophage Cell Factor, Epidermal Cell Derived Thymocyte-Activating Factor, Epidermal Cell Derived Thymocyte Activating Factor, Interleukin I |
| 8 | Interleukin-13 | Interleukin 13 |
| 9 | Interferon-gamma | gamma-Interferon, Immune Interferon, Type II Interferon, Interferon Type II |
| 10 | adiponectin | adiponectin, Adipocyte Complement-Related Protein 30-kDa, Adipocyte Complement Related Protein 30 kDa, Adipose Most Abundant Gene Transcript 1, apM-1 Protein, apM 1 Protein, ACRP30 Protein |
| 11 | CCL2 | Monocyte Chemotactic and Activating Factor, Monocyte Chemoattractant Protein-1, Monocyte Chemoattractant Protein 1, Chemokine CCL2, Chemokine Ligand 2, Monocyte Chemotactic Protein-1, Monocyte Chemotactic Protein 1 |
| 12 | IL-1beta | IL-1b, IL1b, Interleukin-1 beta |
| 13 | prostaglandins | prostaglandin endoperoxides, Prostaglandins a, Prostaglandins b, Prostaglandins d, Prostaglandins e, Prostaglandins f, Prostaglandins i, Prostacyclin |
