# Supplementary material for: Linking Physical Activity to Breast Cancer via Inflammation, Part 2: The Effect of Inflammation on Breast Cancer Risk
Source: Cancer Epidemiol Biomarkers Prev. 2023 Mar 3;32(5):597–605. doi: 10.1158/1055-9965.EPI-22-0929 (PMC10150245; doi:10.1158/1055-9965.EPI-22-0929)
Supplement: Table S2A — Supplementary Table 2A presents the study characteristics of the Mendelian randomization studies [file epi-22-0929_table_s2a_suppst2a.docx]

Supplementary Table 2A: Study characteristics of Mendelian randomization studies

| **Study (Outcome data)** | **Author, year, and country** | **Study Design** | **Participant Characteristics** | **Exposures examined** | **Genetic instrument selection method** |
| --- | --- | --- | --- | --- | --- |
| Breast Cancer Association Consortium (BCAC) | Li 2020, China | Two-sample, summary-level data | 122977 BC cases and 105974 controls of European ancestry | 24 cytokines including CCL2, and IL-13 of interest | Summary statistics for selection on genetic instruments drawn from the Cardiovascular Risk in Young Finns Study. SNPs meeting genome-wide significance threshold kept, SNPs associated with more than 1 cytokine excluded, only SNPs with data available from BCAC included. Only SNPs with strong association to trait were kept. Methods to address linkage disequilibrium employed. |
| Breast Cancer Association Consortium (BCAC) | Robinson 2020, UK | Two-sample, summary-level data | 122977 BC cases and 105974 controls of European ancestry | CRP and 6 adipokines including:  Adiponectin Interleukin-6 Leptin (receptor) | Genetic variants identified in multiple GWAS of individuals of European ancestry. Monogenic or polygenic approach applied to instrument construction, depending on the number of SNPs meeting significance threshold. When few SNPs were available, instruments restricted to cis-variants . |
| Breast Cancer Association Consortium (BCAC) and UK Biobank (UKBB) | Yuan 2020, Sweden | Two-sample, summary-level data | 122977 BC cases and 105974 controls of European ancestry from BCAC; 13666 cases and 353977 controls of European ancestry from UKBB | TNF-a | Conducted a meta-analysis of genome-wide association studies of 25 cohorts encompassing 30912 European descent individuals, chose variants associated with TNF levels at genome-wide significance (P<5 x 10E-8) |
