# Supplementary material for: Linking Physical Activity to Breast Cancer via Inflammation, Part 2: The Effect of Inflammation on Breast Cancer Risk
Source: Cancer Epidemiol Biomarkers Prev. 2023 Mar 3;32(5):597–605. doi: 10.1158/1055-9965.EPI-22-0929 (PMC10150245; doi:10.1158/1055-9965.EPI-22-0929)
Supplement: Table S2B — Supplementary Table 2B presents the study characteristics of the prospective cohort studies [file epi-22-0929_table_s2b_suppst2b.docx]

Supplementary Table 2B: Study characteristics of prospective studies

| **Cohort, country** | **First author and year** | **Study design (follow-up duration); participant characteristics** | **Exposures examined** | **Exposure assessment method** | **Outcome and assessment method** | **Confounding factors used for adjustment** |
| --- | --- | --- | --- | --- | --- | --- |
| EPIC - Varese, Italy | Agnoli 2017 | Nested case-control (14.9 years median); Pre- and post-menopausal women, totals 360 and 334 respectively. 351 women with incident breast cancer and 351 controls. | hsCRP TNF-a IL-6 leptin adiponectin | Blood plasma, using Luminex multiplex technology, levels were grouped into tertiles based on the distribution in controls | Breast cancer (all), obtained through Lombardy Cancer Registry | Age, BMI, family history of breast cancer, age at menarche, parity, oral contraceptive use, education, smoking, alcohol |
| Copenhagen General Population Study, Denmark | Allin 2016 | Cohort (4.8 years median); Pre- and post-menopausal women (age mean 58, IQR 48-67). Cohort of 44,715 participants, of which 822 developed incident breast cancer. Mean age 58 (IQR: 48-67); BMI 25.6 (23.3-28.5) | hsCRP | Blood plasma using standard hospital assays | Breast cancer (all), obtained through Danish Cancer Registry | Age, sex, BMI, Physical Activity, smoking, alcohol consumption, oral contraceptive therapy, hormone replacement therapy |
| Swedish Mammography Cohort, Sweden | Basu 2015 | Nested case-control (Inadequately described; study recruitment between 1987 - 1990; breast cancer cases included until Dec 2011); Postmenopausal women. 69 cases of breast cancer and 719 controls. Age 68.3 (cases), 70.5 (controls); BMI 26.3 | hsCRP | Blood plasma, hsCRP measured using immunoassay with high-sensitivity reagent | Breast cancer (all), histologically confirmed by linkage to Swedish Cancer registers | age at visit, visit date, BMI, height, education, oral contraceptive use, HRT use, age at menopause, parity/age at first birth, family history of breast cancer, benign breast disease, physical activity, alcohol intake, Cathepsin S and Cathepsin B |
| Swedish Mammography Cohort, Sweden | Basu 2016 | Nested case-control (Inadequately described; study recruitment between 1987 - 1990; study published in 2016); Post-menopausal women: 78 cases of breast cancer and 797 controls | PGF2-⍺ | Urinary samples analysed for 8-iso-PGF2a and 15-keto-dihydro PGF2-a by radioimmunoassay. | Breast cancer (all), histologically confirmed by linkage to Swedish Cancer registers | age at blood sampling, date at blood sampling, oral contraceptive use, hormone replacement therapy use, age at menarche, age at menopause, parity/age at first birth, family history of breast cancer, history of benign breast disease, BMI at blood sampling, height, education, physical activity, and alcohol intake |
| Women's Health Initiative, USA | Busch 2018 | Cohort (sample includes participants from a cohort study and a trial) (Analysis censored at 10 years); Post-menopausal women, 394 cases and 10,939 non-cases included in the analysis. Mean age 63, BMI 28 | hsCRP | Blood plasma, high-sensitivity C-reactive protein measured using immunoturbidimetric assay on a Roche/Hitachi Modular P Chemistry Analyzer | Documents such as operative or oncology consultation reports sent from diagnosing clinic to central coordinating centre. | age, race/ethnicity, education, WHI cohort enrolment, age at menarche, age at menopause, parity, amount of time breastfeeding, hormone therapy use, body mass index, smoking, caregiving, negative life events, physical activity, and sleep quality. |
| Shanghai Women's Health Study, China | Cui 2014 | Nested case-control (Median time between sample collection and cancer diagnosis was 4.1 years.); Post-menopausal women. 504 cases and 1,082 controls; | PGE2-M | Urinary prostaglandin E2 metabolite, measured using a liquid chromatography/tandem mass spectrometric method. | Record linkage to Shanghai Cancer Registry | Age, education, smoking, number of live births, moths of breastfeeding. Interaction terms were included in the models to test for interaction between PGE-M and variables of interest (BMI and menopausal status). |
| Northern Sweden Health and Disease Cohort, Sweden | Cust 2009 | Nested case-control (median 4.4 years (time to diagnosis)); 561 cases and 561 controls; 227 cases in women <55 years (assumed pre-menopausal); 334 cases in women >=55 years (assumed post-menopausal). Age categories at diagnosis. | Leptin Adiponectin | Radioimmunoassay | Data linkage to the regional Cancer Registry | Cases and controls matched for aged at baseline and date at blood sampling. Analysis adjusted for BMI and HRT. (Leptin not adjusted for BMI due to high correlation with BMI) |
| Malmö Diet and Cancer Cohort, Sweden | Dias 2016 | Nested case-control (Study recruitment between 1991-1996; follow-up censored at Dec 2010); Post menopausal women at baseline. 446 cases and 885 controls | IL-1b IL-6 IL-8 TNF-a | Analysed with the Human Pro-inflammatory 4-plex II Ultra-sensitive kit | Record linkage to Swedish Cancer Registry and the Southern Swedish Regional Tumor Registry | Age, week of sampling, BMI, hormonal factors (menopausal hormone therapy, parity), abdominal obesity (WHR), smoking status, alcohol, physical activity and education |
| E3N Cohort study, France | Dossus 2014 | Nested case-control (Recruited 1990, censored July 2005); Pre- and post-menopausal women at blood collection, 549 cases, 1040 controls | hsCRP | Particle- enhanced immunoturbidometric assay with lower limit of detection of 0.3 mg/L, intra- assay coefficient of variation \3 %, between-assay \5 %, and functional sensitivity 0.66 mg/L. | Self-reported and verified with medical records review. | Matched on age, menopausal status, date and center at blood collection, and age at menopause. Adjusted for: HRT use, fasting blood sample status, serum sex hormone-binding globulin, estradiol, C-peptide, testosterone, cholesterol and triglyceride levels, or recorded on the questionnaire preceding blood collection, education, age at menarche, oral contraceptive use, parity, number of children, age at first full-term pregnancy, history of benign breast disease, family history of breast cancer in first-degree relatives, smoking status, diabetes, physical activity, total energy intake, and alcohol consumption. Models were then further adjusted for anthropometric measures: BMI , WC, WHR |
| Tromsø, Norway | Frydenberg 2016 | Cohort (14.6 years); Pre- and post-menopausal women. 192 cases 7938 controls | hsCRP | hsCRP was assessed by a particle-enhanced immunoturbidimetric assay | Record linkage to Cancer Registry of Norway | Age at attendance, number of children, body mass index, and current smoking (categorical) |
| Breast Cancer Serum Bank, USA | Gaudet 2010 | Nested case-control (25 years); Post-menopausal women. 234 cases, 234 controls | Adiponectin resistin | Assayed for adiponectin, aPAI-1, and resistin using an endocrine multiplex assay. | Self-reported vital status and cancer diagnoses were supplemented with linkage to the Missouri Cancer Registry (current through May 2003), the Breast Cancer Detection Demonstration Project Cohort files | Cases and controls matched on age and date and time of blood draw. Adjusted for age at reference, body mass index, number of births, age at first full term birth, age at menopause, and current menopausal hormone use. |
| Cancer Prevention Study II Nutrition Cohort, USA | Gaudet 2013 | Nested case-control (15 years); Post-menopausal women. 302 participants with cases and matched controls | CRP  Adiponectin | Measured in serum using analyte-specific, commercially-available ELISA-based assays | Incident cancers diagnosed through June 30, 2007 were self-reported on follow-up questionnaires and subsequently verified by obtaining medical records or through linkage with state registries when complete medical records could not be obtained | cases and controls matched on age and race and adjusted for time from last meal to blood draw, alcohol in the 24 hours before blood draw, prior diagnosis of diabetes, and family history of breast cancer. |
| CLUE-II, USA | Gross 2013 | Nested case-control (25 years); Postmenopausal women. 272 cases, 272 controls | Leptin Adiponectin sTNF-R2 | ELISA assay for all three markers. Leptin using ultrasensitive ELISA. | Incident breast cancer was ascertained by linkage to the Washington County and Maryland Cancer Registries as well as through medical record review and death certificates | Adjusts for age, education, age at menarche, age at first birth, parity, age at menopause, active smoking status, family history of breast cancer, history of breast cysts, alcohol, and recent mammogram |
| Women's Health Initiative Observational Study, USA | Gunter 2015 | Case-cohort (21 years); Post menopausal women. 875 incident cases and 839 subcohort participants. | Leptin adiponectin IL-6 TNF-a CRP | Plasma levels of adiponectin, leptin, TNF-α were analysed using Milliplex Human Adipokine Panels. IL-6 levels were measured using an ultrasensitive solid-phase sandwich ELISA. CRP was assessed with latex-enhanced immunonephelometry. | Breast cancer was identified through annual self-administered questionnaires. Detailed diagnosis were subsequently formally determined through centralized review of medical records. | adjusted for age, ethnicity, alcohol consumption, family history of breast cancer, parity, years of menstrual cycling, age at first child’s birth, type of hormone therapy (HT; in HT users only), endogenous estradiol levels (in non-HT users only), history of benign breast disease, BMI, and physical activity. |
| Nurses Health Study II, USA | Harris 2011 | Nested case-control (6-11 years); Pre- (~80%) and post-menopausal at blood collection. 330 cases, 636 controls | Leptin | Leptin was assayed in luteal and untimed samples by an ELISA that employs a quantitative sandwich enzyme immunoassay technique with a detection limit of 7.8 pg/mL. | Self-reported breast cancer and verification by examination of relevant medical records and pathology reports | Matched on age, menopausal status at blood collection and diagnosis, race/ethnicity, date, time of day and fasting status at blood collection, and luteal day of blood draw (timed cases only). Adjusted for age at menarche, parity/age at first birth, family history of breast cancer, history of benign breast disease, physical activity, BMI at age 18 and weight change from age 18 to blood draw. |
| British Women's Heart and Health Study, UK | Heikkila 2009 | Cohort (About 10 years); Postmenopausal women. 3274 participants were included in the study, including 48 cases of breast cancer | CRP  IL-6 | CRP measured using ultrasensitive nephelometric assay. IL-6 measured using high sensitivity ELISA | Cases were linked with the UK National Health Service Cancer registry | Adjusted for age, BMI, smoking, childhood and adult SEP, physical activity, HRT use, NSAID use |
| Sister Study, USA | Kim 2013 | Case-cohort (2-7 years); Pre- and postmenopausal women. 307 cases, 300 sub-cohort. | Urinary PGE2-M | A major urinary metabolite for PGE2 was quantified using liquid chromatography/tandem mass spectrometry | Self-reported via annual and/or bienniel follow-up questionnaires, or via self-reported diagnosis to study hotline. Diagnosis ascertained through verification of medical records obtained from participants. | Adjusted for regular use of NSAIDs, percent of energy intake from saturated fat, BMI, and family history of breast cancer as covariates. |
| Sister Study, USA | Kim 2017 | Case-cohort (2-7 years); Postmenopausal women. 295 cases, 294 sub-cohort. | Urinary PGE2-M | A major urinary metabolite for PGE2 was quantified using liquid chromatography/tandem mass spectrometry | Self-reported via annual and/or bienniel follow-up questionnaires, or via self-reported diagnosis to study hotline. Diagnosis ascertained through verification of medical records obtained from participants. | Adjusted for age at enrollment, BMI, smoking status, alcohol use status, years of past HRT use, history of breast biopsy, number of first-degree family members with breast cancer |
| Kaiser Permanente Medical Care Program, USA | Krajcik 2003 | Nested case-control (>30years); Pre- and post-menopausal women. Only pre-menopausal results published. 142 participants (71 pairs of matched cases and controls) | TNF-a sTNFR1 sTNFR2 | Blood concentration of TNF-a, sTNFR1 and sTNFR2 were measured and assayed in duplicate using ELISA kits. | Not specified | Adjusted for age and BMI |
| Women's Health Initiative, USA | Nelson 2017 | Cohort (13.6 years mean); Post-menopausal women. 17,841 participants, including 1,114 cases. | hsCRP | Baseline hsCRP was measured using a latex particle–enhanced Immunoturbidimetric Assay Kit. The reference range is 0 to 0.5 mg/dL, and the interassay coefficient of variation was 4.5%. | Outcomes followed-up annually from mailed questionnaires or from non-routine contact by the participant or proxy. Incident cases verified by physician upon medical review. | Adjusted for BMI, race/ethnicity, diabetes, hypertension, smoking status and use of menopausal therapy  Additional variables identified a priori from the literature included physical activity, age, anti-inflammatory use, alcohol use, cardio- vascular disease, and pack-years of smoking were also assessed as confounders but were removed from the model because the effect size change was <10% |
| Multiethnic Cohort, USA | Ollberding 2013 | Nested case-control (Approximately 10 years); Post-menopausal women. 706 Cases and 706 controls. | Leptin adiponectin CRP | ELISA assay for leptin and adiponectin. Latex particle-enhanced turbidimetric assay used to measure CRP | Regular record linkage to Surveillance, Epidemiology and End Results (SEER) cancer registries for Hawaii and California. | Cases and controls were matched on age of birth (1 year), location (HI or CA), ethnicity, date of blood draw (6 months), time of blood draw (2 hours), hours fasting before blood draw (<6, 6–< 8, 8–< 10, !10), and hormone replacement therapy use at blood draw (current vs. not current). Additionally adjust for BMI. |
| Alberta's Tomorrow Project Cohort, Canada | Price 2020 | Cohort (Enrollment between 1987-1989; censored by 31 December 2006); Pre- and post-menopausal women (between 75-76% post-menopausal at baseline). 194 cases and 389 controls. | hsCRP | Immunoturbidimetric assay | Data linkage to the Alberta Cancer Registry (ACR) | Adjusted for age, BF%, total physical activity, smoking status, chronic disease history, family history of breast cancer, menopause status, standing height |
| Mano a Mano Cohort Study, USA | Shen 2019 | Nested case-control (8.2 years median); Pre- and post-menopausal women. 109 breast cancer cases were identified and frequency-matched to 327 controls at a ratio of 1:3, amongst Mexican-American Women. 327 controls | Leptin MCP-1 | Plasma samples were analyzed using Luminex multiplex technology | Annual follow-up phone call and subsequent linkage to the Texas Cancer Registry | Matching criteria were age at recruitment (±2 years), date of biospecimen collection (±1 year), and gender. Adjusted for birthplace, language acculturation, age, parity, body mass index category, education level, smoking status, drinking status, sitting time, and physical activity |
| Rotterdam Study, Netherlands | Siemes 2006 | Cohort (10.2 years median); Post-menopausal women (age >=55 at recruitment). 7,017 participants overall, 184 cases | hsCRP | High-sensitivity CRP measurements were performed using rate near-infrared particle immunoassay | Regional pathology databases were linked to the Rotterdam Study to identify cancer patients in more than 95% of persons within the Rotterdam Study. Followed by physician verification | Adjusted for age, smoking, body mass index, age at menarche and menopause, hormone use, and number of children. |
| Northern Sweden Health and Disease Cohort, Sweden | Stattin 2004 | Nested case-control (1.7 years mean); Post-menopausal women at blood collection. 149 cases, 258 controls | Leptin | Radioimmunoassay | Data linkage to the Swedish Cancer Registry | Cases and controls matched for age, date of blood draw and menopausal status (post-menopausal). Model was adjusted for smoking, insulin and BMI |
| Women's Health Study, USA | Tobias 2018 | Cohort (19 years median); Pre- and Post-menopausal women. Sample size 27,071 participants including 1,497 incident cases | hsCRP | high-sensitivity immunoturbidimetric assay | Mailed questionnaires at 6 months in the first year, annual follow-up thereafter; subsequent confirmation by medical record review by committee of physicians | age, treatment randomization, family history of breast cancer in a relative younger than 60 years of age, personal history of benign breast disease, white race/ethnicity, menopausal status, hormone therapy use, type of most recent hormone therapy use among ever users, age at menarche, parity, age at first birth, oral contraceptive use, mammography screening, Alternative Healthy Eating Index 2010 score, physical activity level, usual frequency of alcohol consumption, smoking, BMI, and other biomarkers |
| SU.Vi.MAX, France | Touvier 2013 | Nested case-control (6.5 years median in cases and 13 years median in controls); Pre- and post-menopausal at baseline. 218 cases, 566 controls. | hsCRP adiponectin leptin MCP-1 | Biomarker levels were determined with an enzyme-linked immunosorbent assay sandwich technique | Self reported and verified with medical records review by independent expert committee | Cases and controls were matched by sex, age, body mass index, and SU.VI.MAX intervention group. Adjusted for age, body mass index, height, SU.VI.MAX intervention group, alcohol intake, physical activity, smoking status, and educational level. |
| Nurses Health Study and Nurses Health Study II, USA | Tworoger 2007 | Nested case-control (Between 4-10 years); Pre- and post- menopausal women. NHS aged 43 to 69; NHS II aged 32 to 54 at blood collection. 1477 cases, 2196 controls | Adiponectin | Adiponectin measured by radio immunoassay | Cases of breast cancer were reported and confirmed by a medical record review, or verbal confirmation by the nurse | Matched on age, menopausal status, HRT use, fasting, time of day and month of blood collection. Adjusted for BMI at age 18, weight change from age 18 to blood draw, family history of breast cancer, history of benign breast disease, duration of PMG use, age of first birth/parity, age at menarche |
| Apolipoprotein Mortality RISk (AMORIS) study, Sweden | VanHemelrijck 2011 | Cohort (Mean 9.74 years for participants without cancer, 5.9 years for participants with cancer (for men and women in the entire cohort)); Pre- and post-menopausal women. 1,241 cases of 55,489 participants | CRP | CRP measured by turbidimetric assay. hsCRP not available | Cases ascertained through data linkage with the Swedish National Cancer Register | Adjusted for age, SES, and history of circulatory disease |
| Nurses Health Study and Women's Health Study, USA | Wang 2015 | Nested case-control (NHS - mean 4.5 years; WHS - mean 8.5 years); Pre- and post- menopausal women (post-menopausal NHS: >70%; WHS: >60%). 2862 cases and 27202 controls | hsCRP | Measured using immunoturbidometric assay, both studies in the same laboratory. Assays were run in two batches in the NHS and one large continuous batch in the WHS. Case–control pairs in the NHS were assayed together and laboratory personnel were blinded to case, control, or quality control status. | NHS - Followed-up biennially to ascertain disease diagnosis. 99% of cases confirmed by medical record review or nurse confirmation. WHS - 6-monthly follow up for the first year, then annual follow-up for self-report diagnosis. Only medical record confirmed cases included | NHS - Adjusted for family history of breast cancer, history of benign breast disease BMI at blood collection, age at menarche, parity and age at first birth, alcohol, smoking, physical activity. Matching variables were age, month of blood collection, time of day of blood draw, fasting status, menopausal status at blood collection, and diagnosis and PMH use at blood collection. WHS - addition of age, randomized treatment assignment, menopausal status and PMH use |
| Kailuan Female Cohort, China | Wang 2015 | Cohort (Between 4-5 years); Pre- and post-menopausal women. Age cut-off at 50 used as proxy for menopausal status <50 years: 10,130; >=50 years: 9,307. Total 19,437 participants and 87 cases. | hsCRP | Levels of hsCRP were measured using high-sensitivity nepelometry assay | Active follow-up of participants by trained physicians, as well as checking of discharge summaries and evaluating medical records from medical insurance. | Adjusted for age, smoking, alcohol, BMI, diabetes, physical activity and marital status |
| Apolipoprotein Mortality RISk (AMORIS) study, Sweden | Wulaningsih 2015 | Cohort (Mean 18.3 years); Pre- and post-menopausal women, breakdown not specified. Mean age 50 (SD 11.56) and 46 (SD 14.78) for cases and non-cases respectively. 6,606 cases of 155,179 participants. | CRP | Turbidimetric assay. No hsCPR available, cut off of 10 mg/L used for dichotomous measure. | Cases ascertained through data linkage with the Swedish National Cancer Register and the Stockholm Clinical Quality Register of Breast Cancer | Adjusted for age, SES, parity, BMI |
| Women's Health Study, USA | Zhang 2007 | Cohort (Mean 10 years); Pre- and Post-menopausal women (between 50-59% post-menopausal at baseline). 27,919 participants in the cohort, of which 892 developed breast cancer | CRP | latex-enhanced immunoturbidimetry | Annual follow-up questionnaire and subsequent confirmation by medical record review | Adjusted for age, randomized treatment assignment, age at menarche, age at first pregnancy lasting 6 months or longer, number of pregnancies lasting 6 months or longer, menopausal status, age at menopause, postmenopausal hormone use, body mass index, family history of breast cancer in mother or a sister, history of benign breast disease, physical activity, multivitamin supplement use, smoking status, and alcohol intake  NSAID adjustment did not significantly change results. |
