# Supplementary material for: Linking Physical Activity to Breast Cancer via Inflammation, Part 2: The Effect of Inflammation on Breast Cancer Risk
Source: Cancer Epidemiol Biomarkers Prev. 2023 Mar 3;32(5):597–605. doi: 10.1158/1055-9965.EPI-22-0929 (PMC10150245; doi:10.1158/1055-9965.EPI-22-0929)
Supplement: Table S3 — Supplementary Table 3 presents the risk of bias assessment, using the ROBINS-E [file epi-22-0929_table_s3_suppst3.docx]

Supplementary Table 3: Risk of bias assessed using the ROBINS-E

| **Study / Cohort** | **Risk of bias item** | | | | | | | **Overall ROB** |
| --- | --- | --- | --- | --- | --- | --- | --- | --- |
|  | **1**  **Confounding** | **2**  **Participant selection** | **3**  **Exposure classification** | **4**  **Exposure departure** | **5**  **Missing data** | **6**  **Outcome assessment** | **7**  **Reporting results** |  |
| EPIC Varese  Agnoli 2017 | Moderate  Adjusted for key confounders | Low | Low  Results with >20% intra-assay variation discarded, sensitivity not reported | Moderate | Low | Low | Low | **Moderate** |
| Copenhagen General Population Study  Allin 2016 | Moderate  Adjusted for key confounders | Low | Moderate  hsCRP, but variation not reported | Moderate | Low  Less than 10% | Low | Low | **Moderate** |
| Swedish Mammography Cohort  Basu 2015 | Moderate  Adjusted for key confounders | Low | Low  Low variation | Moderate  Single biomarker measurement | Moderate  Not reported | Low | Low | **Moderate** |
| Swedish Mammography Cohort  Basu 2016 | Moderate  Adjusted for key confounders | Low | Low  Low variation | Moderate  Single biomarker measurement | Moderate  Not reported | Low | Low | **Moderate** |
| Women's Health Initiative  Busch 2018 | Moderate  Did not adjust for alcohol consumption or diet | Low | Moderate  Sensitivity and variation not reported | Moderate | Moderate  ~90% of controls missing exposure data, but controls were random sample | Low | Low | **Moderate** |
| Shanghai Women's Health Study  Cui 2014 | Moderate  Did not adjust for alcohol or diet | Low | Low  Low variation | Moderate | Low | Low | Low | **Moderate** |
| Northern Sweden Health and Disease Cohort  Cust 2009 | Moderate  Does not adjust for alcohol or diet | Low | Low  Low variation, sensitivity not reported | Moderate | Low | Low | Low | **Moderate** |
| Malmö Diet and Cancer Cohort  Dias 2016 | Moderate  Adjusted for key confounders | Low | Moderate  Inter-assay variation up to 19% for IL-1b, IL-6, IL-8 and TNF-a | Moderate | Moderate  11% missing data  IL-1b was below limit of detection for 74% of samples | Low | Low | **Moderate** |
| E3N Cohort study  Dossus 2014 | Moderate  Adjusted for key confounders | Low | Low  hsCRP, low variation | Moderate | Low  Less than 10% | Moderate  Self-report and verified | Low | **Moderate** |
| Tromsø Cohort Study  Frydenberg 2016 | Moderate  Alcohol not adjusted, did not materially affect results | Low | Low  Low variation | Moderate | Low | Low | Low | **Moderate** |
| Breast Cancer Serum Bank  Gaudet 2010 | Moderate  Only age as confounders did not change results by >15% | Low | Low | Moderate  Two measures, 11% year-to-year reproducibility | Low | Moderate  Self-report and verified, but potential for missing cases. | Low | **Moderate** |
| Cancer Prevention Study II Nutrition Cohort  Gaudet 2013 | Moderate  Adjusts for key confounders | Low | Low  Low variation, high intraclass correlation | Moderate | Low | Moderate  Self-report and verified, but potential for missing cases. | Low | **Moderate** |
| CLUE-II  Gross 2013 | Moderate  Adjusted for key confounders | Low | Low | Moderate | Low | Low | Low | **Moderate** |
| Women's Health Observational Study  Gunter 2015 | Moderate  Adjusted for key confounders | Low | Low  Low variation | Moderate | Low | Low | Low | **Moderate** |
| Nurses Health Study II  Harris 2011 | Moderate  Diet and alcohol not adjusted | Low | Low  Blinded variation test samples 14% variation | Moderate | Low | Moderate  Self-report and verified | Low | **Moderate** |
| British Women's Heart and Health Study  Heikkila 2009 | Moderate  Did not adjust for alcohol consumption or diet | Low | Low  Low variation, hsCRP | Moderate | Low | Low | Low | **Moderate** |
| Sister Study  Kim 2013 | Moderate  Does not adjust for age | Low | Low  Intra- and inter-assay variation 6% and 14%, respectively | Moderate | Low | Moderate  Self-report and verified | Low | **Moderate** |
| Sister Study  Kim 2017 | Moderate  Adjusted for key confounders | Low | Low  Intra- and inter-assay variation 5.2% and 10%, respectively | Moderate | Low | Moderate  Self-report and verified | Low | **Moderate** |
| Kaiser Permanente Medical Care Program  Krajcik 2003 | Moderate  Did not adjust for alcohol consumption or diet | Low | Moderate  Intra- and inter-assay variation 14.3% and 18.7%, respectively | Moderate | Low | Moderate  Outcome measurement methods not reported | Low | **Moderate** |
| Women's Health Initiative  Nelson 2017 | Moderate  Alcohol not adjusted as change in estimate was <10% | Low | Low  Low variation | Moderate | Low | Moderate  Self-report and verified | Low | **Moderate** |
| Multiethnic Cohort  Ollberding 2013 | Moderate  Did not adjust for alcohol as it did not materially affect estimate. | Low | Low  Low variation | Moderate | Moderate  Missing values not reported | Low | Low | **Moderate** |
| Alberta's Tomorrow Project Cohort  Price 2020 | Moderate  Alcohol and diet did not change estimates | Low | Moderate  Variation and sensitivity not reported | Moderate | Low | Low | Low | **Moderate** |
| Atherosclerosis Risk in Communities (ARIC)  Prizment 2013 | Moderate  Did not adjust for alcohol consumption or diet | Low | Low | Moderate | Moderate  Missing values not reported | Low | Low | **Moderate** |
| Mano a Mano Cohort Study  Shen 2019 | Moderate  Adjusted for key confounders | Low | Low  Samples >10% of intra-assay variation were re-analyzed | Moderate | Low | Low | Low | **Moderate** |
| Rotterdam Study  Siemes 2006 | Moderate  Did not adjust for alcohol as it did not materially affect estimate. | Low | Moderate  Did not report sensitivity or other assay validity measures | Moderate | Low  Multiple imputation | Low | Low | **Moderate** |
| Northern Sweden Health and Disease Cohort  Stattin 2004 | Moderate  Did not adjust for alcohol consumption or diet | Low | Low | Moderate | Low | Low | Low | **Moderate** |
| Women's Health Study  Tobias 2018 | Moderate  Adjusted for key confounders | Low | Low  Very low variation | Moderate | Low  Missing indicator categories used | Moderate  Self-report and verified | Low | **Moderate** |
| SU.Vi.MAX  Touvier 2013 | Moderate  Adjusted for key confounders | Low | Low  Variation measures <10% | Moderate | Moderate  Missing BMI (covariate) data in ~30% of cases | Moderate  Self-report and verified | Low | **Moderate** |
| Nurses Health Study and Nurses Health Study II  Tworoger 2007 | Moderate  Adjusts for key confounders | Low | Low  Intra-assay variation at 7-13% | Moderate | Low | Low | Low | **Moderate** |
| Apolipoprotein Mortality RISk (AMORIS) study  VanHemelrijck 2011 | Serious  Does not adjust for BMI, alcohol or diet | Low | Moderate  hsCRP not available, variation 12% | Moderate | Moderate  Not reported | Low | Low | **Serious** |
| Nurses Health Study and Women's Health Study  Wang 2015 | Moderate  Adjusted for key confounders | Low | Low  Moderate intra-assay variation at 7-15% | Moderate | Low | Moderate  Self-report and verified | Low | **Moderate** |
| Kailuan Female Cohort  Wang 2015 | Moderate  Adjusted for key confounders | Low | Low  hsCRP, Low variation between 4-6%. | Moderate | Low | Low | Low | **Moderate** |
| Apolipoprotein Mortality RISk (AMORIS) study  Wulaningsih 2015 | Moderate  Adjusts for key confounders | Low | Moderate  Intra-assay variation 12% Unable to measure concentrations <10mg/L | Moderate | Low | Low | Low | **Moderate** |
| Korean Cancer Prevention Study-II  Yoo 2018 | Moderate  Adjusted for key confounders | Low | Low | Moderate | Low | Low | Low | **Moderate** |
| Women's Health Study  Zhang 2007 | Moderate  Adjusted for key confounders | Low | Moderate  No sensitivity, or inter- or intra-assay variation reported. | Moderate | Low | Low | Low | **Moderate** |
